# Non-metastatic 2 (NME2)-mediated suppression of lung cancer metastasis involves transcriptional regulation of key cell adhesion factor vinculin

**DOI:** 10.1093/nar/gku860

**Published:** 2014-09-23

**Authors:** Ram Krishna Thakur, Vinod Kumar Yadav, Akinchan Kumar, Ankita Singh, Krishnendu Pal, Luke Hoeppner, Dhurjhoti Saha, Gunjan Purohit, Richa Basundra, Anirban Kar, Rashi Halder, Pankaj Kumar, Aradhita Baral, MJ Mahesh Kumar, Alfonso Baldi, Bruno Vincenzi, Laura Lorenzon, Rajkumar Banerjee, Praveen Kumar, Viji Shridhar, Debabrata Mukhopadhyay, Shantanu Chowdhury

**Affiliations:** 1 Proteomics and Structural Biology Unit, Institute of Genomics and Integrative Biology, CSIR, Mall Road, Delhi 110 007, India; 2 G.N.R. Knowledge Centre for Genome Informatics, Institute of Genomics and Integrative Biology, CSIR, Mall Road, Delhi 110 007, India; 3 Academy of Scientific and Innovative Research (AcSIR), New Delhi, India; 4 Department of Biochemistry and Molecular Biology, Mayo Clinic College of Medicine, Rochester, MN, USA; 5 Animal House, Centre For Cellular and Molecular Biology, Uppal Road, Hyderabad 500 007, India; 6 Department of Biochemistry, Section of Pathology, Second University of Naples, Italy; 7 University Campus Biomedico, Rome, Italy; 8 Department of Surgery, University La Sapienza, Rome, Italy; 9 Division of Lipid Science and Technology, Indian Institute of Chemical Technology, Hyderabad, India; 10 Department of Experimental Pathology, Mayo Clinic Cancer Center, Rochester, MN, USA

## Abstract

Tumor metastasis refers to spread of a tumor from site of its origin to distant organs and causes majority of cancer deaths. Although >30 metastasis suppressor genes (MSGs) that negatively regulate metastasis have been identified so far, two issues are poorly understood: first, which MSGs oppose metastasis in a tumor type, and second, which molecular function of MSG controls metastasis. Herein, integrative analyses of tumor-transcriptomes (*n* = 382), survival data (*n* = 530) and lymph node metastases (*n* = 100) in lung cancer patients identified non-metastatic 2 (NME2) as a key MSG from a pool of >30 metastasis suppressors. Subsequently, we generated a promoter-wide binding map for NME2 using chromatin immunoprecipitation with promoter microarrays (ChIP-chip), and transcriptome profiling. We discovered novel targets of NME2 which are involved in focal adhesion signaling. Importantly, we detected binding of NME2 in promoter of focal adhesion factor, vinculin. Reduced expression of NME2 led to enhanced transcription of vinculin. In comparison, NME1, a close homolog of NME2, did not bind to vinculin promoter nor regulate its expression. In line, enhanced metastasis of NME2-depleted lung cancer cells was found in zebrafish and nude mice tumor models. The metastatic potential of NME2-depleted cells was remarkably diminished upon selective RNA-i-mediated silencing of vinculin. Together, we demonstrate that reduced NME2 levels lead to transcriptional de-repression of vinculin and regulate lung cancer metastasis.

## INTRODUCTION

More than 90% of cancer-related mortality results from metastasis (1,2). Metastatic lesions are manifested in multiple steps, including localized invasion and intravasation at the primary tumor site, sustained survival in circulation, extravasation at distant organ site(s) and colonization at the new site (3,4). It is intriguing that despite the complexity, a set of genes known as metastasis suppressor genes (MSGs) can negatively regulate the metastatic process (5). Although > 30 MSGs have been identified so far in various tumor types (reviewed in (6,7,8)), two issues remain poorly understood: first, the specificity of known MSGs for a tumor type, and second, how does reduced expression of MSG contribute to metastasis.

Primary tumors in lung metastasize to brain, bone, contra-lateral lung, liver and kidney, and metastatic growths are a major cause of cancer-related mortality in men and women worldwide (9,10). In this report, we identified a key MSG, NME2, from a pool of >30 MSGs through analysis of tumor transcriptomes, overall patient survival data and lymph node metastases in lung cancer patients. We investigated the molecular mechanisms of NME2 function through genomic, cellular and molecular studies, including model systems and validation in tumor/metastatic lymph node tissue obtained from lung cancer patients. Findings for the first time reveal that a MSG regulates a key focal adhesion factor to control lung cancer cell dissemination.

## MATERIALS AND METHODS

### Cell lines and culture conditions

A549 cell line was obtained from the American Type Culture Collection. Cell line was authenticated by the cell bank with short tandem repeat analysis. The cells were expanded and stored according to the supplier's instructions and used within 6 months of recovery of frozen aliquots. The cells were maintained in Dulbecco's modified Eagle medium supplemented with 10% fetal bovine serum at 37°C in 5% CO_2_ environment. Details of generation of stable cell lines are provided in the supplementary data.

### ChIP-chip, peak generation, motif discovery, transcription factor enrichment

ChIP assays were performed as per the protocol provided by Upstate Biotechnology. Three independent experiments were performed, cross-linked chromatin was immunoprecipitated, sequenase amplified, labeled, fragmented and DNA hybridized to a set of Affymetrix 1.0 R oligonucleotide microarrays. A 12-mer motif for NME2 was identified by Gibbs motif sampler at its default parameters. The peak sequences were given as input into the Match tool to scan for enriched PWMs listed in TRANSFAC professional.

### Cancer cell extravasation assays in zebrafish

Control, vinculin-depleted, NME2-depleted and vinculin, NME2 double knockdown A549 cells were trypsinized, counted and labeled with Cell Tracker Orange CMTMR (Invitrogen, Carlsbad, CA, USA) according to the manufacturer's instructions. The A549 cells were resuspended in phosphate buffered saline containing DNase I and heparin, and 50–200 cells were microinjected into the pericardium of anesthetized 3 days-post-fertilization Tg(Fli-GFP) zebrafish. The zebrafish were put in 37°C embryo water (water supplemented with salts for maintaining zebrafish embryos) for 24 h following injection and imaged on a ZEISS LSM 780 confocal microscope using standard FITC and dsRed filter sets.

### Tail vein metastasis assays in nude mice

Approximately 1.5×10^6^ A549 cells were injected into tail vein per mice; lungs were stained for human vimentin (Millipore Inc. Massachusetts, USA) 8 weeks post-injection. Collagen was stained by vWF antibody (Chemicon/Millipore Inc. Massachusetts, USA). Confocal images were taken using Lieca TCS SP5 (Leica, Germany). The animal usage and protocols involved were performed according to the guidelines approved by the Institutional Animal Ethical Committee of IICT and CCMB, Hyderabad.

### Clinical annotation of tumor specimen

For analysis of expression of NME2 and VCL, we used commercially available lung tumor samples (cDNA qRT PCR arrays from Origene, Inc., USA). The primary lung tumors and matched lymph node metastases for immunohistochemical (IHC) analysis of NME2 and VCL were from the archives of the Anatomic Pathology of the Second University of Naples, Italy (period from 1993 to date). The details on age, sex and pathology are provided as part of the supplementary data.

### IHC staining

Antibodies against vinculin were from Abcam, USA. For Immunohistochemistry, primary lung tumor and autologous lymph node metastases were stained for NME2 (antibody from Kamiya Biomedical Company, Seattle, USA) and counterstained for haematoxylin.

### Statistical analysis

*In vitro* cellular assays were done at least in triplicate, and *P*-values were derived using student's t test unless stated otherwise. The error bars represent standard deviation unless described otherwise. Statistical significance for immunostaining of NME2 in primary tumors and autologous lymph node metastases was calculated using Pearson chi-square test.

Additional description of Materials and Methods is provided in the supplementary data.

## RESULTS

### Integrative approaches to prioritize key MSGs associated with malignant progression of lung tumors

To identify the key MSGs dictating metastasis of lung tumors, we used a three-tiered approach: (i) identification of MSGs which showed consistently reduced expression in advanced tumors relative to early stage tumors across independent studies; (ii) analysis of association between expression levels of MSGs and overall patient survival and (iii) whether MSG expression correlated to lymph node metastasis in patients.

**Figure 1.**
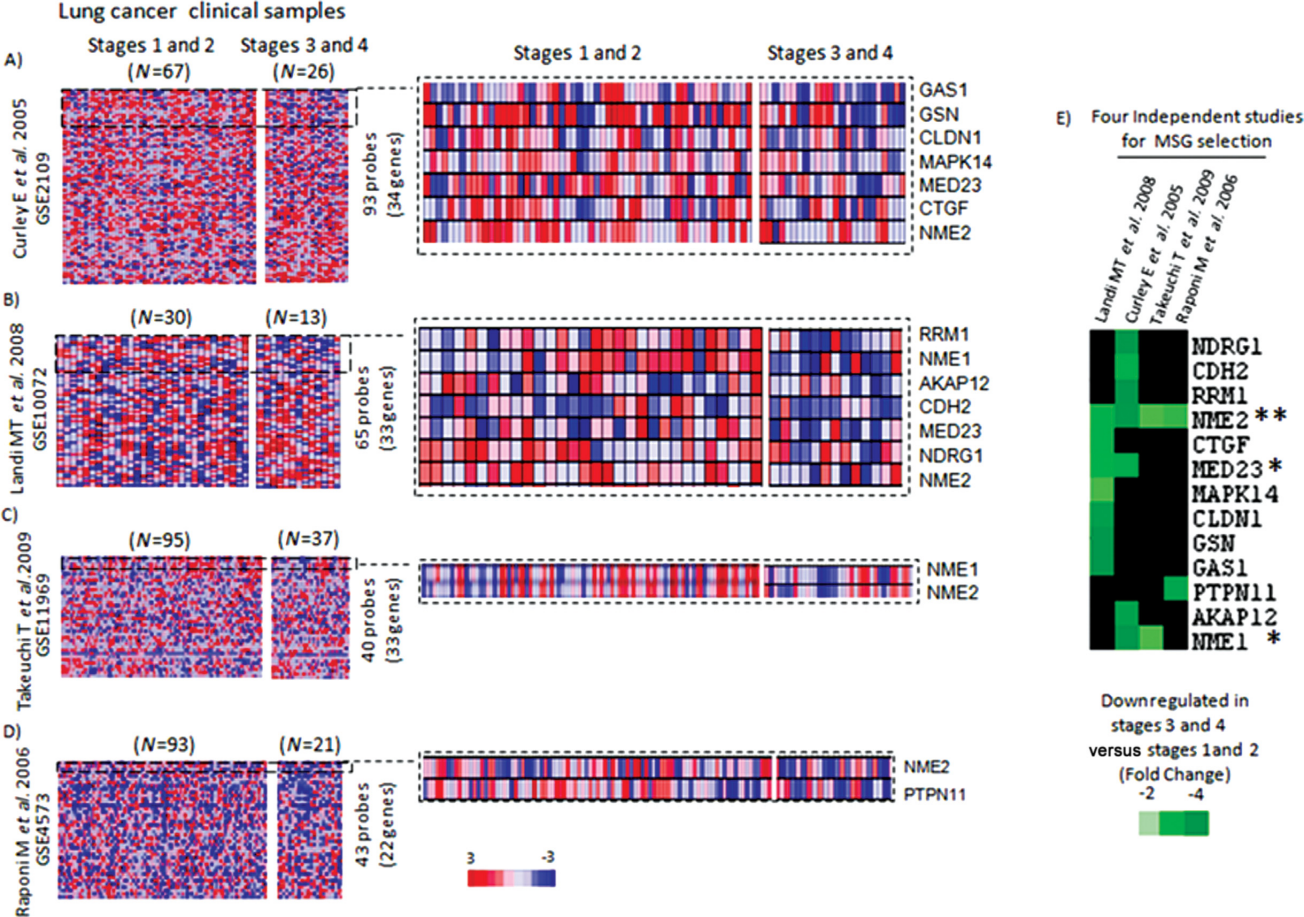
Large-scale analyses of tumor transcriptomes identify key MSG in lung cancer metastasis. Analysis of differential expression of >30 MSGs across four independent datasets (A–E); **(A)** representation of analysis from Curley *et al.* (expression project in Oncology (expO)), **(B)** Landi *et al.*, **(C)** Takeuchi *et al.* and **(D)** Raponi *et al*. The number of MSGs for which probes were found in individual study is also indicated next to the heat maps. Projections from individual heat maps show changes in expression of MSGs; the right panel indicates subset of MSGs that showed reduced expression in advanced stages relative to early stages; expression index: red: upregulation, blue: downregulation. **(E)** A heat map for 13 MSGs that showed reduced expression in advanced tumor stages in at least one dataset examined; A fold change of −2, −3 and −4 corresponds to 50%, 66% and 75% decrease, respectively, in expression level compared to early stage tumors; * = gene with reduced expression in at least two datasets; ** = gene with reduced expression in all four datasets. Scale: fold change of expression.

Transcriptomes from lung cancer patients (*n* = 382) were grouped according to stage in each individual study: advanced (III and IV) versus early (I and II). Our analyses revealed an interesting pattern: out of >30 MSGs (Supplementary Table S1), 13 had reduced expression in at least one study; only two MSGs, NME1 and MED23, had reduced expression in two studies, and remarkably, only a single factor, NME2 (or NM23 H2), was found to have significant reduced expression across all the four studies (Figure 1A–E).

### Increased NME2 correlates with enhanced overall survival in lung cancer patients

**Figure 2.**
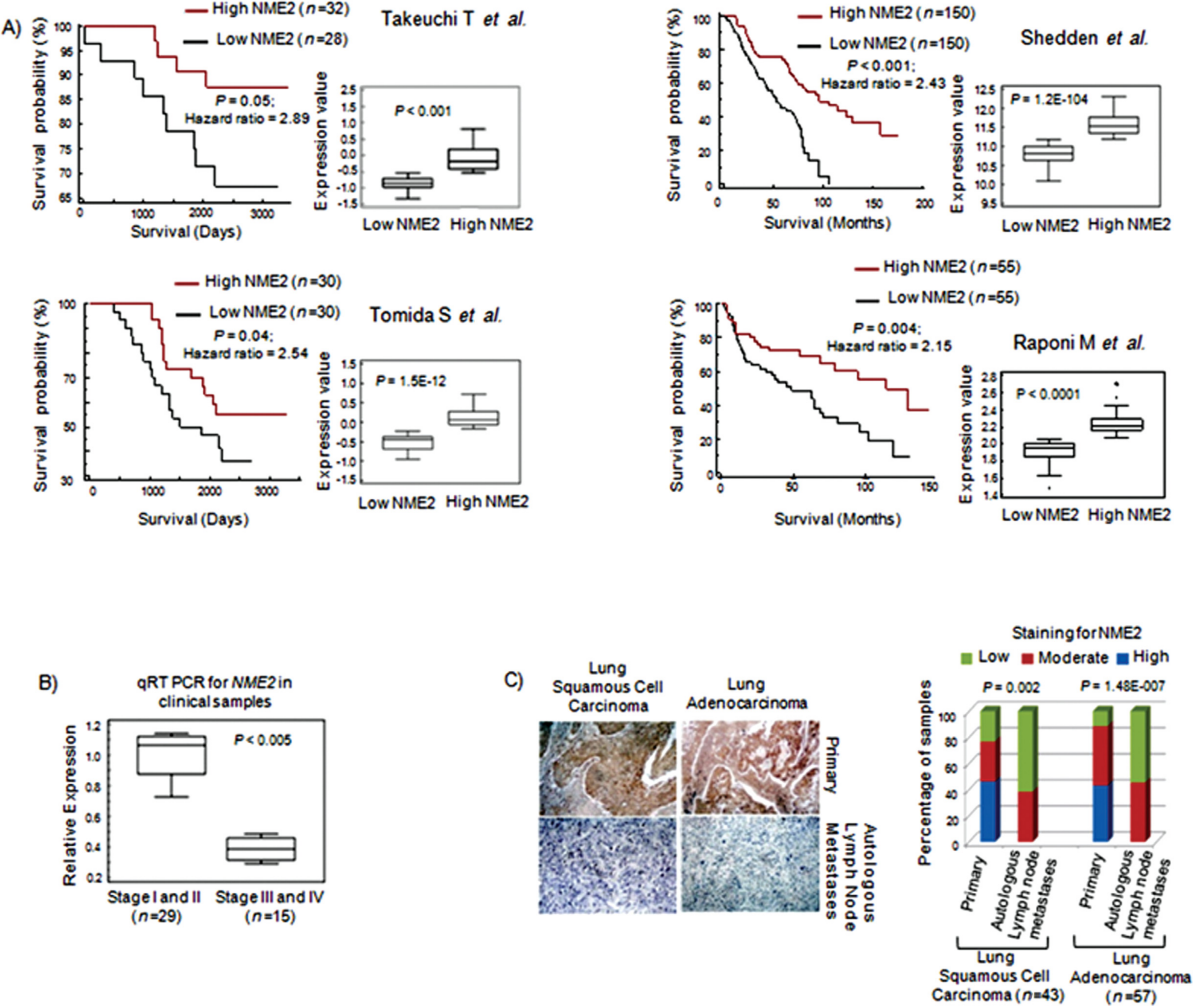
Metastasis suppressor NME2 expression associates with overall survival and autologous lymph node metastasis in patients. Kaplan–Meier plot was used to analyze relationship between NME2 transcript level and patient survival in data from **(A)** Takeuchi T *et al.*; *n* = 60, Shedden *et al.*; *n* = 300, Tomida *et al.*; *n* = 60 and Raponi *et al.*; *n* = 110; hazard ratio for each analysis shown. Inset in all cases shows box plot of NME2 transcript level in the two groups tested; statistical significance was calculated using student's t test. **(B)** qRT PCR for *NME2* mRNA in 44 tumors grouped stage-wise from lung cancer patients; 1.7-fold depletion corresponds to 41% decreased expression compared to early stage tumors. **(C)** Representative immunohistochemistry images for NME2 in primary lung tumors and autologous lymph node metastases (left); quantitation of NME2 immunostaining (right panel, statistical significance calculated by Pearson chi-square test).

We next analyzed which of the three identified MSGs (NME2, MED23 and NME1) correlated with overall patient survival. Using four independent datasets on overall patient survival (*n* = 530), we first grouped patient samples based on high versus low expression of individual MSG and then evaluated association between MSG expression and overall patient survival. Analysis of datasets from Takeuchi *et al.* (*n* = 60) (11), Shedden *et al.* (*n* = 300) (12), Tomida *et al.* (*n* = 60) (13) and Raponi *et al.* (*n* = 110) (Figure 2A) showed increased expression of NME2 correlated with enhanced overall survival relative to low NME2 levels (Figure 2A). Using similar analyses for both NME1 and MED23 in all four datasets, we found that NME2 expression showed the best correlation with overall patient survival (Supplementary Figure S1A–D).

Next patient tumor DNA and tissues were examined for expression of NME2 during progression from early to advanced stages, and lymph node metastases. Quantitative real-time (qRT) PCR for NME2 showed significantly reduced transcript level in advanced stage tumor samples (*n* = 44, ∼1.7-fold depletion; equivalent to 41% decrease, *P* < 0.003; Figure 2B) confirming the trend observed in tumor transcriptome meta-analyses. This result was further supported by IHC analysis of NME2 in primary lung tumors and matched lymph node metastases from same patients (*n* = 100; adenocarcinoma as well as squamous cell carcinoma) where we found reduced NME2 level in metastases compared to primary tumors (Figure 2C).

### Transcriptome profile of NME2-depleted cells correlates with profiles obtained from advanced lung tumors and implicates control of focal adhesion pathway

**Figure 3.**
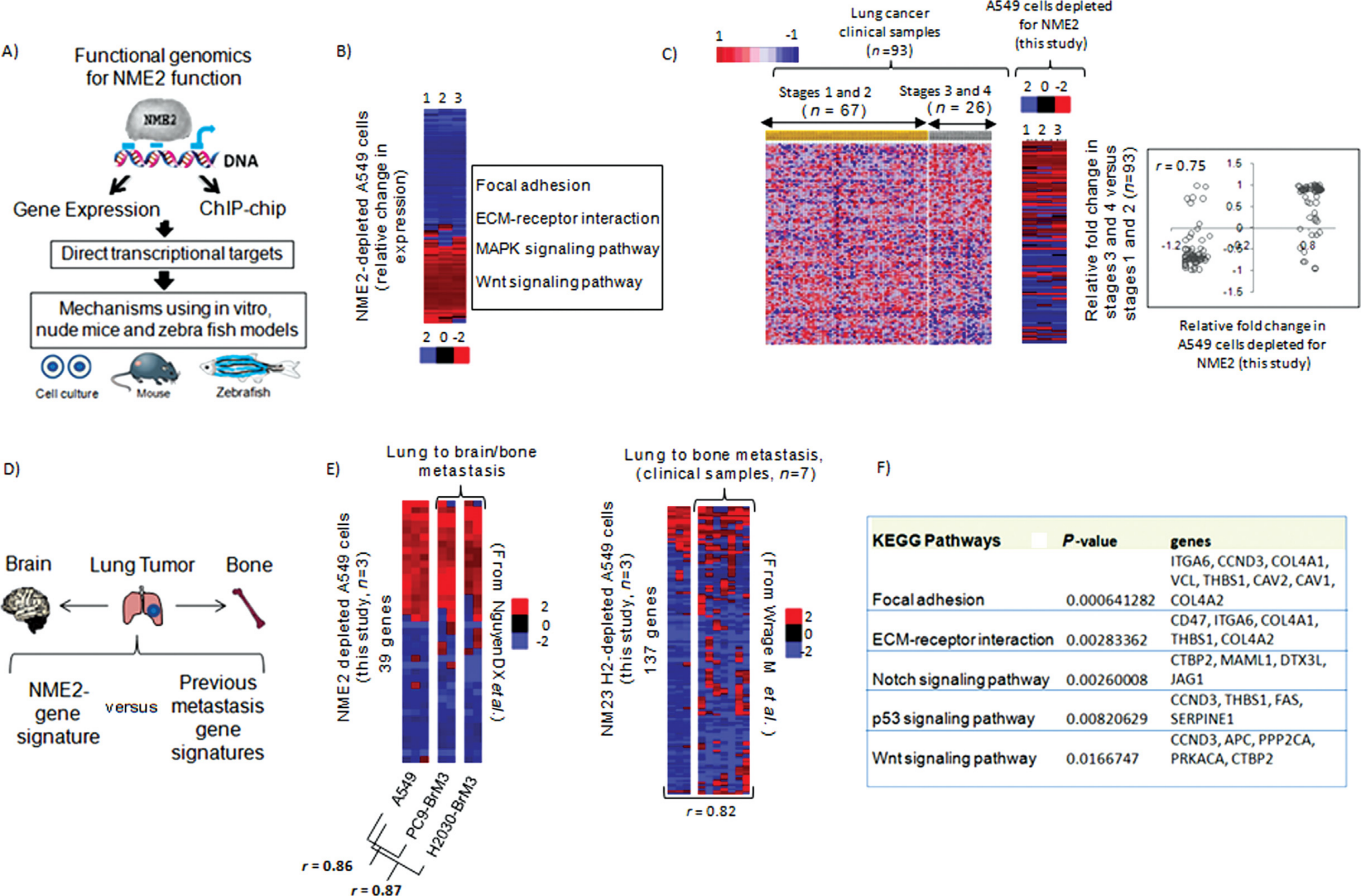
NME2 depletion results in gene expression changes characteristic of tumors with metastatic proclivity. **(A)** A functional genomics framework for exploring and validating functions of key metastasis suppressor, NME2; use of gene expression microarrays and chromatin immunoprecipitation coupled to hybridization to promoter microarrays (ChIP-chip) to identify direct targets, and use of *in vitro* and *in vivo* models for assessing contribution of targets to cancer progression. **(B)** Gene expression changes on NME2 depletion in A549 lung adenocarcinoma cells: heat map of 296 genes showing relative change in expression (1–3 are biological replicates); functional classification of the genes shows enriched biological pathways using Gene Ontology (GO)—top four KEGG pathways are shown. Expression change after depletion of NME2 in A549 cells relates closely to advanced lung cancers. **(C)** Left panel: heat map of normalized expression profile of 101 genes (126 probes) across 93 clinical datasets grouped stage-wise (*P* < 0.01); right panel: correlation of relative fold change in expression between clinical samples and NME2-depleted A549 cells (correlation coefficient, *P* < 0.001). **(D)** Primary lung tumors metastasize mainly to bones and brain; a framework to compare NME2-gene expression signature with previous organ-specific metastasis gene signatures. **(E)** Expression profile of NME2-depleted A549 cells relates to primary lung adenocarcinomas with preference for metastasis to bone and brain; heat maps represent relative fold changes in gene expression from Subramanian *et al.* (21; see the text), and Nguyen *et al.* (23; see the text) in left and right panel, respectively (PC9-BrM3 and H2030-BrM3 are lung cancer cells with demonstrated metastatic proclivity to both brain and bone, respectively). **(F)** Focal adhesion genes show deregulated expression in lung tumors with metastatic proclivity; GO of genes shared between NME2-depleted A549 cells and lung cancer cells with metastatic preference for bone and brain.

NME2 belongs to NME (non-metastatic 23/NM23) gene family, which are the first discovered metastasis suppressors (14). Among NME members, NME1 and 2 have been most studied in the context of metastasis suppression (15,16). However, only NME2 showed consistently reduced expression in advanced lung tumors (Figure 1A–E) and significantly associated with overall patient survival (Figure 2A and Supplementary Figure S1). Therefore, we focused on NME2 in this study. Several studies have implicated NME2 as a regulator of genes implicated in cancer progression such as c-MYC, PDGF-A, ITGAM (17,18,19) and telomerase (20). Therefore, we asked whether the gene regulatory roles of NME2 were important in the control of metastasis and adopted a combinatorial strategy of (i) gene expression profiling and (ii) chromatin immunoprecipitation coupled to promoter microarray hybridization (ChIP-chip) to identify novel targets of NME2 and subsequently assessed their contribution to metastasis (Figure 3A).

First, in order to understand whether lung adenocarcinoma-derived A549 cells represent a possible model for molecular studies, we probed the transcriptome of A549 cells following NME2 depletion and assessed its correlation with the gene expression profile of patient-derived lung tumors. Investigation of >47,000 transcripts following si-RNA-mediated NME2 depletion in A549 cells revealed 296 genes as differentially regulated (124 genes upregulated and 172 downregulated *P* < 0.005, Figure 3B, left panel; 29 out of 30 genes were validated by qRT PCR, False Discovery Rate (FDR) <5%; Supplementary Table S2). These 296 genes were designated as ‘NME2-responsive genes’. Gene ontology (GO) analysis of NME2-responsive genes using Genecodis 2 (21,22) identified focal adhesion as the most significant pathway (Figure 3B, right panel).

Next, we compared the expression pattern of NME2-responsive genes in A549 cells with lung cancer clinical samples (*n* = 93) grouped as advanced (III and IV) versus early stage (I and II). We used gene set enrichment analysis (GSEA), a widely adopted computational method for determination of statistical differences in gene expression between the chosen biological states (21). We found that 101 genes (126 probes) showed a pattern of differential expression in lung cancer clinical samples that significantly correlated with the expression change noted in A549 cells following depletion of NME2; 44 genes were upregulated and 57 genes downregulated in advanced compared to early stages (Figure 3C).

As primary lung tumors frequently metastasize to brain and bone (22), we checked whether gene expression program of NME2-depleted cancer cells compared to transcriptomes of brain and bone metastases (Figure 3D). We observed significant similarity between transcriptomes of NME2-depleted A549 cells and lung adenocarcinoma with demonstrated preference for metastatic proclivity to bone and brain (23,24) (Figure 3E). Importantly, the genes common to three studies showed significant enrichment of focal adhesion pathway (Figure 3F). The analyses supported the findings made above (Figure 3B) that NME2-mediated metastatic suppression might involve regulation of key genes of focal adhesion pathway.

### Combinatorial analysis of promoter-wide binding and gene expression reveals novel targets of NME2

**Figure 4.**
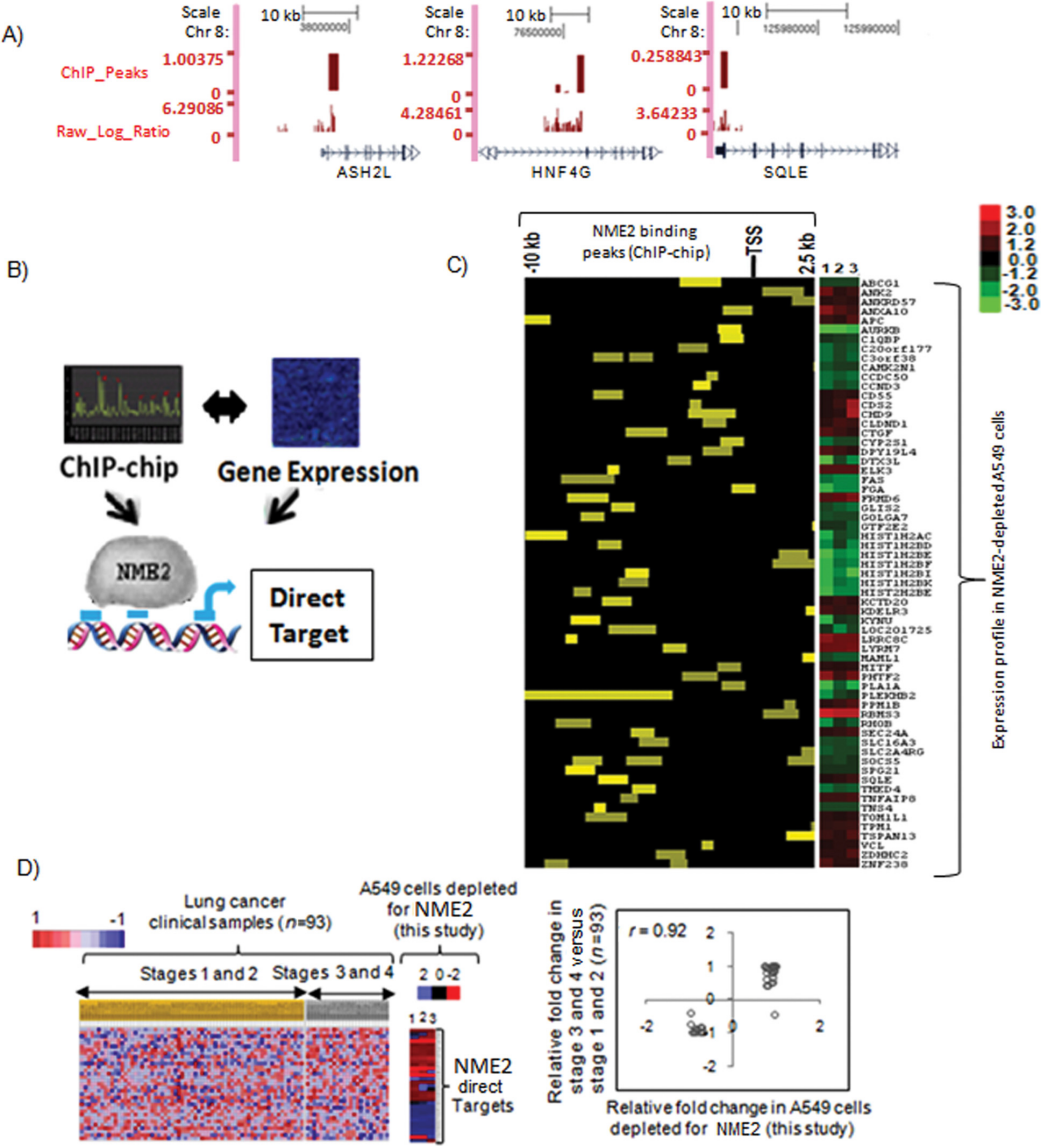
Promoter-wide location analysis identifies targets of NME2 involved in metastatic progression. **(A)** ChIP-chip for NME2 across promoters of human genome: representative binding sites (peaks) and enrichment score on chromosome 8 in UCSC browser format. **(B)** Overlap of ChIP-chip and expression microarray results identified genes with change in mRNA level as well as binding of NME2 to their respective promoters as direct targets of NME2. **(C)** Position of NME2 binding site with respect to TSS and ChIP-chip enrichment score within the 64 direct targets of NME2 (left panel) and the relative change (log ratio) in expression of the transcript as determined by microarrays on targeted depletion of NME2 in A549 cells (right panel). **(D)** NME2-direct targets (36 probes representing 27 genes) show changes in expression across 93 lung carcinoma transcriptomes (grouped stage-wise) concordant with changes in gene expression profile obtained in A549 cells; *P* = 0.003 (left panel); correlation of relative fold change in expression in clinical samples versus NME2-depleted A549 cells (*P* < 0.0001 for correlation coefficient) (right panel).

We used ChIP-chip to identify NME2 binding sites across promoters of the human genome and found a total of 553 binding peaks of NME2 at >2-fold binding enrichment (*P* < 0.001, Figure 4A); 372 binding peaks out of 553 localized on 346 unique promoters (typically within −7.5 kb to +2.5 kb of transcription start sites (TSS)) suggesting that some promoters had more than one binding peak for NME2. We found that 21 out of 22 promoters tested showed enrichment in ChIP-PCR (FDR of <5%, Supplementary Figure S2A; Supplementary Table S3). Using the Gibbs motif sampler (25) we found a guanine-rich 12-mer sequence motif present in > 80% of NME2 ChIP-chip peaks, which is largely consistent with earlier reports of NME2 as a purine-binding factor (Supplementary Figure S2B) (17,18,19). Furthermore, using position weight matrices for transcription factor binding sites reported in TRANSFAC database (25) we found binding sites of six transcription factors: PAX4, PLZF, POU3F2, PAX6, OG2 and PPAR gamma to be significantly enriched within NME2 binding peaks (Supplementary Figure S2C).

As promoters differ in their chromatin architecture, it is expected that not all promoters would show a change in transcription. Therefore, we defined NME2-direct targets as the genes, which changed their expression upon NME2 depletion and also harbored NME2 binding peaks within respective promoters (Figure 4B, left panel). Combined analysis of ChIP-chip peaks at an enrichment score of 1.2-fold and transcriptome analysis significance (at *P* < 0.005) resulted in identification of 64 genes (30 upregulated and 34 downregulated; Figure 4C, right panel) as NME2 direct targets. Thus, while NME2 occupied 346 gene promoters, 64 of these promoters showed change in transcription on NME2 depletion. Several of the target genes were validated by ChIP-PCR (Supplementary Figure S2A) and qRT PCR for expression (Supplementary Table S2). GO of NME2 direct target genes showed distinct involvement in regulation of transcription, nucleosome assembly, apoptosis and notably cell adhesion (Supplementary Figure S2D; *P* < 0.05 in all cases). Among genes related to cell adhesion, we found vinculin *(VCL)*, adenomatous polyposis coli (*APC*), Rho related GTP binding protein B (*RHO B*) and connective tissue growth factor (*CTGF*) as direct targets of NME2. Together with NME2-responsive genes found to be enriched for focal adhesion (Figure 3B and F), these genes act at different levels to control cell adhesion and signaling (26).

In order to understand the role played by NME2-regulatory network in metastasis, we asked whether expression of the 64 NME2 target genes related to progression of lung cancer. Analysis of lung cancer gene expression datasets using GSEA revealed that 27 genes (33 probes), which showed differential expression between advanced (III and IV) versus early stages (I and II), were significantly correlated with expression change in NME2-depleted A549 cells; 16 genes were upregulated and 11 downregulated (Figure 4D). This suggested that regulatory potential of NME2 is closely linked to progression into advanced cancers with metastatic proclivity.

### NME2 transcriptionally represses focal adhesion factor vinculin

**Figure 5.**
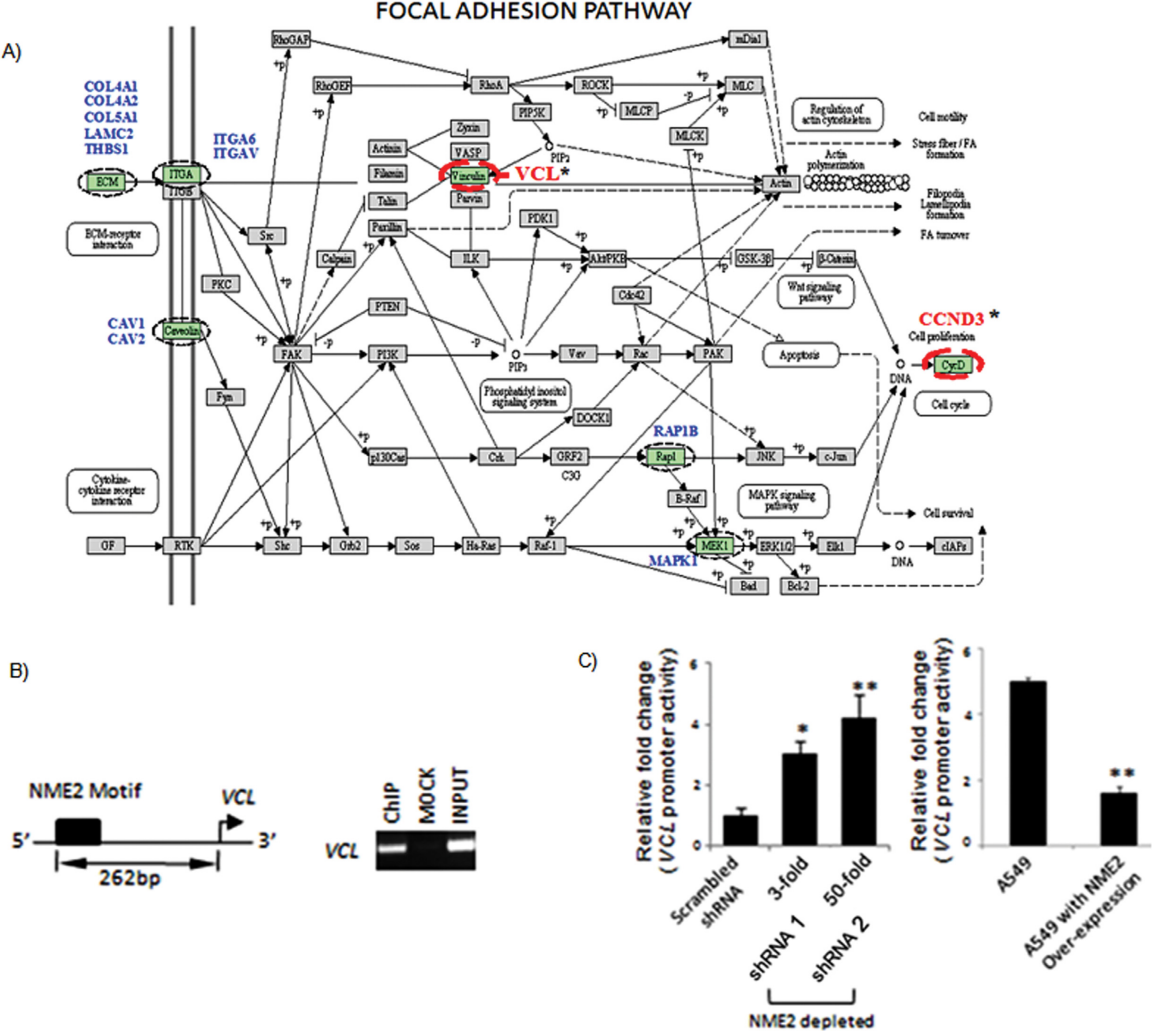
Focal adhesion pathway gene vinculin as a direct transcriptional target of metastasis suppressor NME2. **(A)** Representation of NME2-target genes enriched in KEGG focal adhesion pathway; names are mentioned against highlighted boxes. Genes in blue color showed changes in mRNA level only (indirect targets); in comparison, genes with asterisk (*) showed alteration in mRNA level and binding of NME2 to their promoter as well (direct targets). **(B)** ChIP-PCR validation of NME2 occupancy of vinculin promoter: scheme showing NME2-motif within vinculin promoter; ChIP assay shows NME2 occupancy at the vinculin promoter; mock represents immunoprecipitation using non-specific isotypic IgG. **(C)** Over expression of NME2 represses (left panel), and stable depletion of NME2 enhances vinculin promoter activity in a luciferase reporter assay (right panel); 3- and 50-fold depletion corresponds to 66% and 98% decreased expression compared to control; all significance values: *P < 0.05, **P < 0.01; Student's t test, error bars represent standard deviation (SD).

Our analyses so far implicated regulation of focal adhesion pathway by NME2; first, we found changes in transcript level of genes of focal adhesion pathway in response to NME2 in our gene expression microarray analysis (Figure 3B for microarray and Figure 5A for representation), and second, comparison of NME2-gene signature with previous metastasis signatures showed the highest enrichment of focal adhesion pathway (Figure 3F). Interestingly, we noted NME2 occupancy within the promoter of vinculin, a key component of the focal adhesion pathway (27) (Figure 4C) and further tested the relationship between NME2 and vinculin. We found a 12-mer motif for NME2 located 262 bases upstream of vinculin TSS (Figure 5B, and Supplementary Figure S2B for motif sequence). This was also validated independently using ChIP for NME2 followed by PCR with primers that flanked the motif (Figure 5B). Our observations prompted us to investigate whether NME2 could directly bind to vinculin promoter. We employed a direct binding assay where 12-mer oligonucleotide forming the NME2-motif was used to check *in vitro* binding with purified recombinant NME2 (a mutated version of the nucleotide with key residues substituted served as control; details in supplementary materials and methods). Use of direct binding assay clearly showed that purified NME2 protein binds to vinculin promoter, and the binding was sharply diminished when NME2-motif was disrupted (Supplementary Figure S3). Interestingly, NME1, a homolog of NME2, did not show specific binding to vinculin promoter suggesting a transcription regulatory role specific for NME2 (Supplementary Figure S3).

Furthermore, we noted increased expression of vinculin upon NME2 suppression from analysis of microarrays (Figures 3B and 4C), and this was independently confirmed at the mRNA and protein level (Supplementary Figure S5; see Supplementary Figure S4 for stable depletion of NME2; different shRNAs were used to generate stable cell clones). In line, endogenous mRNA levels of vinculin decreased when NME2 was expressed in A549 cells (Supplementary Figure S5). In addition, this was further confirmed by luciferase reporter assays. The vinculin promoter harboring the NME2-motif showed increased promoter activity upon NME2-depletion in stable lines, conversely, the vinculin promoter activity decreased upon NME2 expression (Figure 4C, and Supplementary Figure S4 for generation of A549 cells with stable depletion of NME2). We also probed the contribution of kinase deficient mutant of NME2 (19) in regulation of vinculin promoter activity. We found that similar to wild-type NME2, kinase mutant also suppressed activity of vinculin promoter (Supplementary Figure S6A). This is consistent with our earlier observations where we had noted that kinase dead mutant did not affect transcription (19). Furthermore, we investigated the effect of NME1 on vinculin promoter activity in luciferase assays; our results showed that NME1 did not significantly alter activity of vinculin promoter (Supplementary Figure S6B). In line, targeted silencing of NME1 did not lead to any change in endogenous mRNA level of vinculin (Supplementary Figure S6C and D). Together, these results demonstrated that NME2 negatively regulates vinculin expression through control of vinculin promoter activity, and this activity is not exhibited by NME1.

### Metastatic potential of NME2-depleted lung cancer cells is attenuated in absence of vinculin *in vivo*

**Figure 6.**
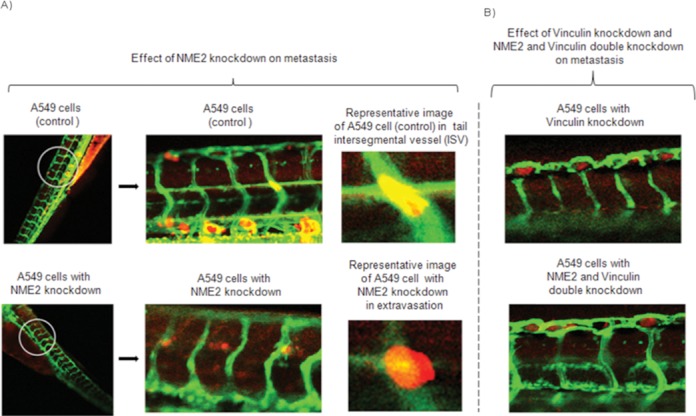
NME2 dictates extravasation of cancer cells through its target vinculin in zebrafish model of metastasis. Effect of knockdown of NME2 and vinculin on extravasation of A549 cells in zebrafish model of metastasis (A–B); following injection of A549 cell clones (red) into pericardium of 3 dpf Tg(Fli-GFP) zebrafish, the imaging was done 24 h later using a confocal microscope. In Tg(Fli-GFP) zebrafish, vasculature expresses GFP and appears green. **(A)** Control A549 cells (red) are visible within the circulatory system (green) of zebrafish and have not extravasated. A549 cells with NME2 knockdown (red) are extravasating from the circulatory system (green) and shown in extravascular space. Representative image of a control A549 cell in the tail ISV of a zebrafish (top) and an image of an extravasating NME2-depleted A549 cell (bottom). **(B)** A549 cells with vinculin knockdown (red) and NME2 as well as vinculin double knockdown (red) are visible within the circulatory system (green) of zebrafish and none have extravasated. Dpf: days-post-fertilization.

We next examined whether NME2 depletion could promote metastasis *in vivo* (in zebrafish and immunocompromised mice), and whether its target, vinculin, was required for NME2-mediated control of metastasis. Zebrafish is increasingly becoming accepted as a particularly attractive model system for studying metastasis (28). Indeed, because zebrafish embryos are optically transparent, these lend themselves well to study of engrafted cancer cells by confocal microscopy (28). We performed a qualitative assay utilizing an *in vivo* cancer metastasis extravasation model (29) to assess the metastatic potential of NME2-depleted, vinculin-depleted, and NME2 and vinculin double knockdown A549 cells in zebrafish (Supplementary Figure S7 for generation of double knockdown cells). A549 cells were labeled with a red tracker dye and microinjected into the pericardium of 3 day post-fertilization (dpf) Tg(fli-GFP) zebrafish embryos; pericardium represents the most relevant site of injection as it enables cancer cells to directly enter into circulation (29). After 24 h, we observed NME2-depleted cells in the extravascular space, whereas control cells as well as vinculin-depleted and NME2–vinculin double knockdown cells remained in the intersegmental vessels (ISV) (Figure 6A). Furthermore, we found NME2-depleted A549 cells actively extravasating from the ISV, but no similar extravasation events were seen in zebrafish injected with the control scrambled shRNA, vinculin-depleted or NME2–vinculin double knockdown cells (Figure 6B). As the A549 cells with scrambled shRNA control did not show any extravasation, it is clear that the present experimental design did not detect any artifactual movement of cells unrelated to metastasis. Together, these findings demonstrate that while NME2 depletion promoted metastasis, loss of vinculin diminished metastatic potential of NME2-depleted cells. We next probed if NME2 depletion promoted metastatic dissemination of lung cancer cells in an experimental model of metastasis in nude mice. We found 2-fold enhanced metastatic seeding by NME2-depleted cells relative to control A549 cells (Supplementary Figure S8A and B; *n* = 5 in each case, *P* < 0.05). These results supported the observations made in zebrafish and demonstrated that reduced expression of NME2 promoted metastasis by A549 cells *in vivo*.

Increased invasiveness and resistance to anoikis are features of aggressive cancers (4,30). In line with our findings, NME2-depleted A549 cells consistently displayed enhanced invasiveness across extracellular matrix (ECM) compared to control cells (Supplementary Figure S9A). As expected, increased percentage of NME2-depleted A549 cells survived under anoikis-inducing conditions compared to control cells (Supplementary Figure S9B). Furthermore, similar to *in vivo* observations in zebrafish, targeted silencing of vinculin in NME2-depleted A549 cells diminished their invasiveness across ECM (Supplementary Figure S9C and D). Furthermore, vinculin depletion in NME2-depleted A549 cells perturbed downstream signaling as evident by decrease in phospho-paxillin (Supplementary Figure S9E). Collectively, these results demonstrate that NME2-mediated regulation of vinculin favors a signaling pathway that contributes to invasiveness and metastasis of cancer cells.

### Increase in NME2 correlates to reduced vinculin expression in lung tumors

**Figure 7.**
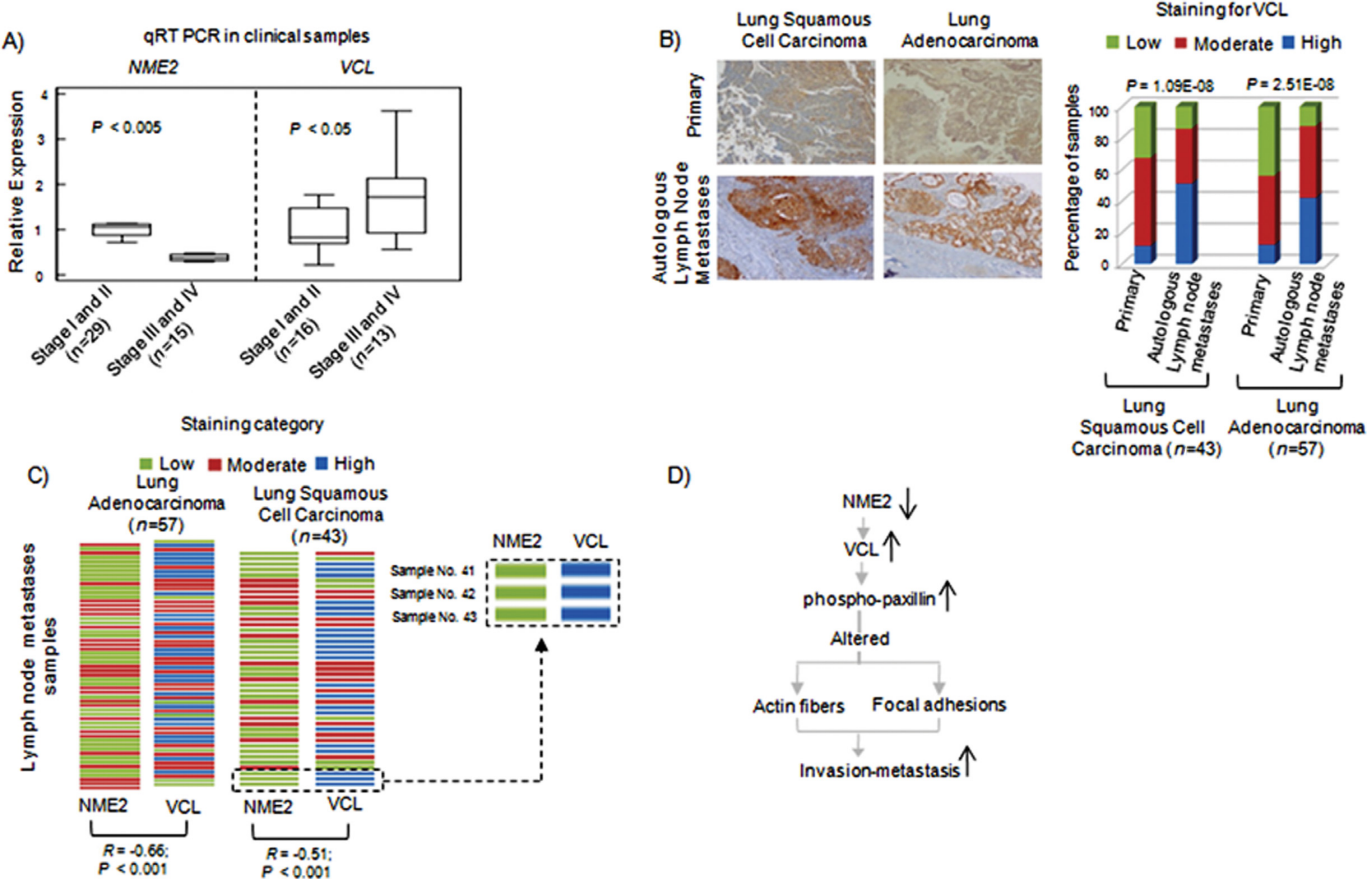
Inverse relationships between vinculin and NME2 expression in lymph node metastases. **(A)** qRT PCR for NME2 and vinculin transcript in stage-wise grouped tumors from lung cancer patients. Significance calculated by student's t test. **(B)** Representative images of IHC staining for vinculin in lung tumors (left) and quantitation of vinculin immunostaining (right). **(C)** Matched staining results for both NME2 and Vinculin in lymph node metastases of 100 patients; inset shows staining pattern for NME2 and vinculin in three patients; strength of staining shown by color (top). **(D)** A proposed model for anti-metastatic function of NME2 involving regulation of vinculin.

qRT PCR analysis showed significantly lower NME2 expression in advanced lung tumors (Figure 2B). Examination of vinculin transcripts in lung tumors showed the opposite trend: higher in advanced stages, clearly demonstrating an inverse relationship between NME2 and vinculin (*n* = 29; *P* < 0.05; Figure 7A, right panel). Next, we probed level of vinculin in primary tumors versus matched lymph node metastases (derived from same patients, *n* = 100) through IHC staining. This analysis showed increased vinculin expression in metastases relative to primary tumors (Figure 7B). We compared the expression of NME2 and vinculin in a pair-wise fashion in lymph node metastases (*n* = 100) by assigning a score to intensity of IHC staining for each (Figure 7C). An inverse relationship between NME2 and vinculin expression was clear from this analysis; metastases were marked by low level of NME2 but high level of vinculin, as noted in A549 cells. Importantly, to validate our observation in different lung cancer lines, we found increased vinculin levels upon NME2 knockdown in H157 cells, another non-small cell lung carcinoma line (Supplementary Figure S10). Based on these findings, we propose a model of metastasis control (Figure 7D) in which reduced expression of NME2 in tumor cells perturbs focal adhesion signaling by enhancing expression of vinculin. Increased vinculin acts as a molecular trigger for altered cytoskeletal rearrangement and enables metastasis.

## DISCUSSION

Although a correlation between reduced expression of NME members and micro-dissemination of lung tumors has been noted (31,32) as well as an association between decreased levels of NME2 transcripts with advanced stages of lung tumors (33), the prognostic significance of NME2 in the context of lung cancer progression has been doubted (34,35). Correspondingly, direct demonstration of NME2 action as a metastasis suppressor in lung cancer has been lacking. Herein, our functional analyses in zebrafish and nude mice showed that reduced expression of NME2 promotes metastatic dissemination of lung cancer cells. Furthermore, our results provide a molecular framework for metastasis suppression by NME2, which involves regulation of vinculin. Recent findings suggest that an enhanced level of vinculin is necessary for generating focal adhesion contacts at the cell periphery which impart contractile forces for invasion through 3D matrix of extracellular proteins (27). A signaling cascade downstream of vinculin involving phosphorylation of cytoskeletal adaptors (paxillin) and kinases, including focal adhesion kinase and myosin light chain kinase, has been implicated in determination of cellular invasiveness through acto-myosin cycling (27). In line with these observations, we found that the metastatic potential of NME2-depleted A549 cells in zebrafish (*in vivo*) and invasiveness through basement membrane matrix (*in vitro*) was diminished upon depletion of vinculin. This supports requirement of vinculin downstream of NME2 in regulation of cell invasion and metastasis.

Apart from vinculin, our findings show a large number of NME2-specific association sites within promoters (346 in total). For example, previous studies have demonstrated NME2-mediated regulation of *c-myc* (17,18,19). Recently, NM23 occupancy on 10 promoters was observed (however, the study did not discriminate between NME1 and NME2) (36). Despite these reports, the full regulatory potential of NME2 has remained in doubt and unexplored because first, separate DNA binding and trans-activation domains have not been characterized thus far for NME2, and second, NME2 was shown to interact with single stranded nucleotides rather non-specifically (please see the detailed description in the supplementary data) (37,38). However, our ChIP-chip results showed specific binding of NME2 with target promoters revealing its transcriptional potential. In line, our direct binding assays demonstrated that NME2 directly binds to its target promoters such as vinculin unlike its homolog, NME1. The magnitude of change in VCL promoter activity (using luciferase reporter assays) was similar in case of NME1 or NME2 expression (∼20% increase or decrease, respectively). In contrast, on targeted silencing we observed distinctly different results in case of NME1. NME1 silencing resulted in no detectable (by real-time PCR) difference in VCL expression (Supplementary Figure S6C and D), whereas in case of NME2 silencing there was >40% increase in VCL mRNA (Supplementary Figure S5). Taken together with direct DNA binding results showing specific binding of NME2, and not NME1 to the vinculin promoter element (Supplementary Figure S3), we inferred that NME1 does not bind to the vinculin promoter and nor does it regulate vinculin.

Interestingly, recent observations suggest that NME2 may be part of transcriptional complexes and work in tandem with other transcription factors such as OCT1 (39) or Estrogen receptor beta (40). Notably, we observed association of NME2 with histone 2B promoter in our ChIP-chip data (Figure 4C). This is consistent with a previous finding which also showed NME2 localization at the H2B promoter as part of OCA-S transcriptional co-activator complex (39). Thus, while the precise mechanism of NME2 association with DNA may be currently unknown, it is clear from the results obtained in this study that NME2 can influence the gene regulatory program of cancer cells.

Our observations indicate that NME2-mediated gene expression may be crucial to metastasis. For example, we found that NME2 associates with promoters of transcription factors: ELK3, GLIS2, GTF2E2, c-MYC, MITF, SLC2A4RG, ZNF238 and CHD9 (Figure 4C). Several of these genes have been implicated in metastasis regulation via control of Wnt/beta-catenin signaling and response to hypoxia and tumor angiogenesis (41,42). NME2 depletion led to upregulation of *ELK3*, which has been independently shown to regulate HIF-alpha and enhance angiogenesis (41). Interestingly, GLIS2, another regulatory target of NME2, has been demonstrated to function as a negative regulator of Wnt/beta-catenin signaling pathway and was crucial for lung cancer metastasis to brain (23,42).

To our knowledge, this is the first study of the regulatory network of NME2, a key MSG with transcription-related function. Using a combination of genomic approaches such as ChIP-chip, we identify novel targets of NME2 and demonstrate that NME2–vinculin signaling is important for metastasis *in vitro* and *in vivo*. Therefore, therapeutic approaches that target NME2–vinculin signaling by either increasing NME2 expression or decreasing vinculin level in lung tumors could be of potential clinical significance. It is noteworthy that several MSGs, such as BRMS1, CRSP3, TXNIP, KDM1A and KLF17, apart from NME2 possess transcription-related functions (Supplementary Table S1), and therefore the integrative approaches used in the study could be of interest to researchers working on these genes as well.

## ACCESSION NUMBERS

The gene expression and ChIP-chip data have been submitted to Gene Expression Omnibus (http:/www.ncbi.nlm.nih.gov/geo/) under accession number GSE18285.

## SUPPLEMENTARY DATA

Supplementary Data are available at NAR Online.

SUPPLEMENTARY DATA
