# Supplementary material for: Non-metastatic 2 (NME2)-mediated suppression of lung cancer metastasis involves transcriptional regulation of key cell adhesion factor vinculin
Source: Nucleic Acids Res. 2014 Sep 23;42(18):11589–600. doi: 10.1093/nar/gku860 (PMC4191424; doi:10.1093/nar/gku860)
Supplement: SUPPLEMENTARY DATA [file supp_42_18_11589__index.html]

Non-metastatic 2 (NME2)-mediated suppression of lung cancer metastasis involves transcriptional regulation of key cell adhesion factor vinculin — Non-metastatic 2 (NME2)-mediated suppression of lung cancer metastasis involves transcriptional regulation of key cell adhesion factor vinculin — SUPPLEMENTARY DATA 

# Non-metastatic 2 (NME2)-mediated suppression of lung cancer metastasis involves transcriptional regulation of key cell adhesion factor vinculin

## SUPPLEMENTARY DATA

**Files in this Data Supplement:**

- SUPPLEMENTARY DATA
