# Supplementary material for: Non-metastatic 2 (NME2)-mediated suppression of lung cancer metastasis involves transcriptional regulation of key cell adhesion factor vinculin
Source: Nucleic Acids Res. 2014 Sep 23;42(18):11589–600. doi: 10.1093/nar/gku860 (PMC4191424; doi:10.1093/nar/gku860)
Supplement: SUPPLEMENTARY DATA [file supp_gku860_nar-00662-x-2014-File009.doc]

**Supplementary Data**

**Supplementary Figures, Tables, Materials and Methods and Clinical Information**

**Non-metastatic 2 (NME2)-mediated suppression of lung cancer metastasis involves transcriptional regulation of key cell adhesion factor vinculin**

Thakur et al. 2014

The gene lists and GO classification are available at <http://quadbase.igib.res.in/NME2_ChIP_on_chip>

**Supplementary Figure S1**

**
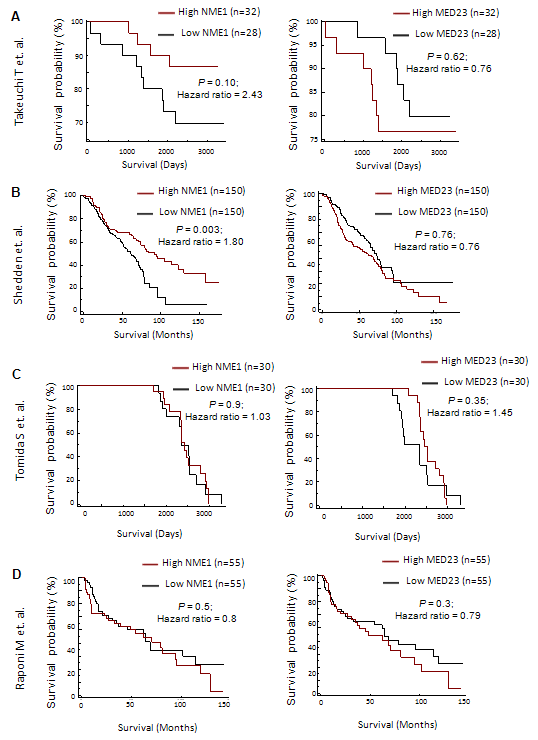
**

**Figure S1 Survival analysis for NME1 and MED23**

**A-D**. Kaplan-Meier plot was used to analyze relationship between NME2 transcript level and patient survival in data from **(A)** Takeuchi T et al. Journal of Clinical Oncology, 2006; n=60, **(B)** Shedden et al. Nature Medicine, 2008; n=300, **(C)** Tomida S et al. Journal of Clinical Oncology, 2009; n=60, and **(D)** Raponi et al. Cancer Research, 2006; n=110; hazard ratio for each analysis shown. Statistical significance was calculated using student’s t test.

**Supplementary Figure S2**

**
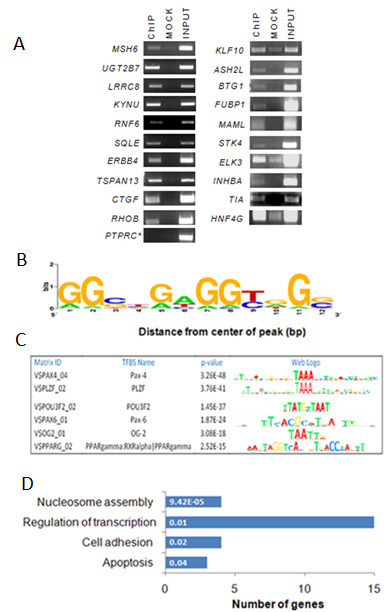
**

**Figure S2 NME2 ChIP-chip analysis**

**A.** Semi quantitative PCR of 20 representative promoter regions to validate ChIP-chip results; PTPRC* is a negative control locus. **B.** WebLogo of the NME2 motif within binding sites found by ChIP-chip. **C.** Occurrence of transcription factor binding sites with significant enrichment within NME2 ChIP-chip peaks analyzed using TRANSFAC. **D.** Gene ontology for 64 direct transcriptional targets of NME2, Gene ontology classification of 64 NME2-direct targets which changed expression (up regulated and down regulated) following targeted NME2- depletion was performed using Genecodis 2.

**Supplementary Figure S3**

**
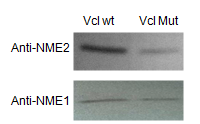
**

**Supplementary Figure S3** **Direct binding assay to check interaction of NME2 and NME1 to vinculin promoter**

One microgram of streptavidin agarose beads linked to biotinylated oligonucleotide probes from 12-mer NME2 motif in vinculin promoter was incubated with recombinant NME2 (upper panel; 1 micro gram) and NME1 (lower panel; 1 micro gram) in binding buffer (15% Glycerol, 12mM HEPES, 4mM Tris, 150mM KCl, 1mM EDTA and 1mM DTT). Western blotting using antibody from Abcam, Cambridge, UK (Abcam - ab60602) which recognizes both NME1 and 2 with similar efficiency was used to analyze the precipitates. The wild type sequence of oligonucleotide from vinculin promoter is the following: biotin-5’-CTCCTTATAAG^*^G^*^GCATAATGGGAGCGGAGCGG; the sequence which is underlined represents 12-mer NME2 motif; the bases marked by * were mutated. Mutant oligonucleotide sequence: biotin- 5’-CTCCTTATAAAAGCATAATGGGAGCGGAGCGG; mutant/disrupted oligonucleotide served as binding control. Additional bases flanking 12-mer motif were added to allow for efficient biotinylation. A detailed description of direct binding assay conditions appears as part of supplementary materials and methods under heading: ‘direct binding assay’.

**Supplementary Figure S4**

**
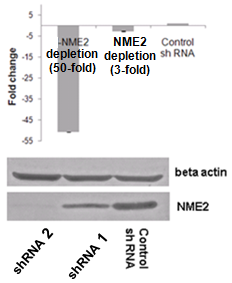
**

**Figure S4 Generation of cells with stable depletion of NME2**

Generation of A549 cells with stable depletion of NME2 expression for use in cellular assays and nude mice experiments; NME2 expression, mRNA (quantitative real time PCR) (upper panel) and protein levels (lower panel) of A549 cells was observed following partial depletion of NME2 (with ~3-fold reduced expression at mRNA level using shRNA 1; corresponds to ~66% decreased from control) or almost total depletion of NME2 (with ~50-fold reduced expression at mRNA level using shRNA 2; corresponds to ~98% decrease from control) using NME2-specific shRNA 1 and 2. Note that different shRNAs were used for generating the cell clones (details are provided in supplementary information under the heading: "Generation of stable NME2-depleted, and NME, vinculin double knockdown clones of A549 cells and viability analysis").

**Supplementary Figure S5**

**
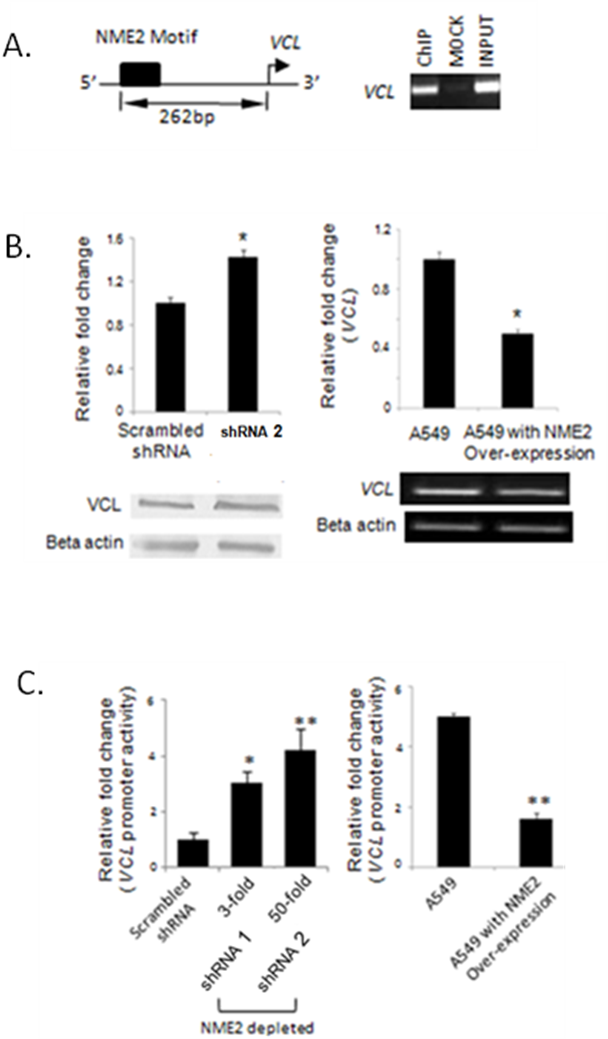
**

**Figure S5 NME2 depletion increases while NME2 over expression decreases vinculin level respectively.**

Western blot and densitometric estimation of increase in vinculin protein level (left) and relative difference in transcript levels obtained from PCR (right); shRNA 2 resulted in reduced expression at mRNA level of NME2 by ~50-fold corresponding to ~98% decrease from control. All significance values: * P<0.05, Student’s t test, error bars represent standard deviation (S.D.).

**Supplementary Figure S6**

**
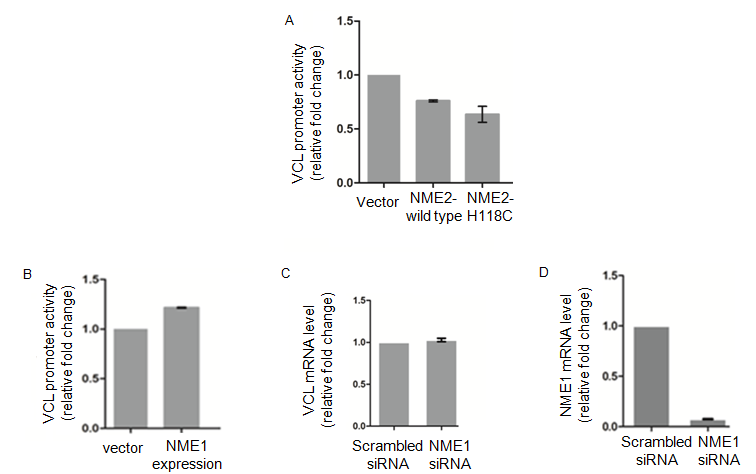
**

**Supplementary Figure S6 Comparison of effects of NME2 and NME1 on vinculin promoter activity**

**A.** Measurement of vinculin promoter activity in presence of wild type and kinase mutant NME2. Luciferase reporter incorporating vinculin promoter was used in presence of NME2 (wild type), and nucleoside diphosphate kinase deficient (Histidine 118^th^ substituted with cysteine). Reporter assays were performed at least thrice. **B.** Influence of NME1 on vinculin promoter activity in presence of wild type NME1. **C.** vinculin mRNA expression was checked upon targeted silencing of NME1 by real time PCR. **D.** NME1 was silenced using NME1 specific siRNA; results depict reduction in NME1 mRNA level as measured by real time PCR.

**Supplementary Figure S7**

**
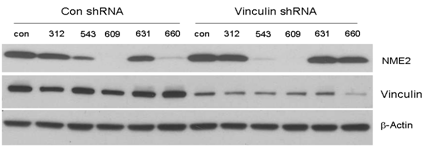
**

**Figure S7 Generation of cells with double knockdown of NME2 and vinculin**

Generation of cells with NME2 and vinculin double knockdown for use in zebrafish experiments; First vinculin was knocked down in A549 cells using lentiviral shRNA and then five different lentiviral clones of NME2 shRNA (labeled in figure as 312, 543, 609, 631 and 660) were used to knock down NME2 in both control (without vinculin depletion) and vinculin depleted cells. With shRNA 609 maximal NME2 knock down was achieved, and this clone was used for further experiments in zebrafish.

**Supplementary Figure S8**

**
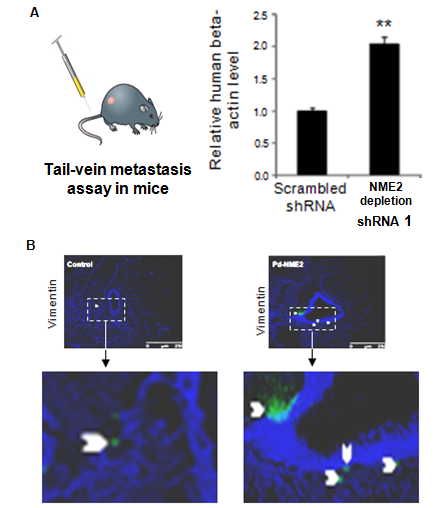
**

**Figure S8 NME2-depleted cancer cells seed metastasis efficiently**

Human lung adenocarcinoma A549 cells stably depleted for NME2 seed metastases in nude mouse model; **A.** Quantification of metastasis measured by expression of human beta actin relative to mouse beta actin in treated/untreated mice lungs (qRT PCR; n=5, *P*< 0.05, student’s t test); shRNA 1 resulted in reduced expression at mRNA level of ~3-fold corresponding to ~66% decrease from control. **B.** Staining of lung cryosections from treated mice using human specific vimentin antibody for tumor cells**.** Nuclei were stained with 4, 6-diamidino-2-phenylindole (DAPI). All significance values: ** P<0.01; Student’s t test, error bars represent standard deviation (S.D.)

**Supplementary Figure S9**

**
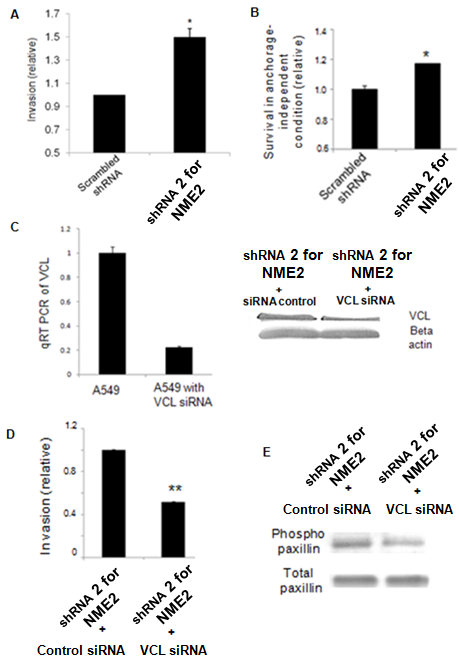
**

**Figure S9 NME2 mediated regulation of vinculin controls invasiveness**

**A.** Characterization of invasiveness of A549 cells stably depleted for NME2 across membrane coated with extracellular matrix proteins; **B.** Measurement of anchorage-independent survival following depletion of NME2 in A549 cells. **C.** Targeted depletion of VCL level using siRNAs directed against VCL (qRT PCR analysis and western blot; left and right panel respectively). **D.** Targeted reduction of vinculin decreases invasiveness of NME2-depleted A549 cells. **E.** Vinculin depletion leads to decreased level of phospho-paxillin; western blot for phospho- and total paxillin; shRNA 2 resulted in reduced expression at mRNA level of ~50-fold corresponding to ~98% decrease from control. All significance values: * P<0.05, ** P<0.01; Student’s t test, error bars represent standard deviation (S.D.).

**Supplementary Figure S10**

**
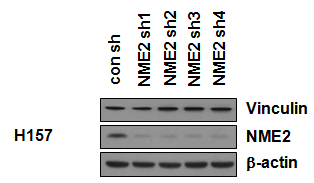

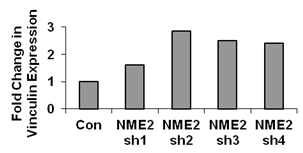
**

**Figure S10 shRNA mediated depletion of NME2 leads to increased vinculin expression in H157 cells.**

Vinculin expression (at the protein level in the left panel; densitometric analysis in the right panel) in human non-small cell lung carcinoma H157 cells following depletion of NME2 by individual shRNAs (sh1 to sh4 represent different shRNAs commecially available; detailed information in supplementary methods under the heading: "Generation of stable NME2-depleted, and NME, vinculin double knockdown clones of A549 cells and viability analysis"). Expression of NME2, vinculin and beta actin was probed at the protein level. Beta actin served as the loading control.

**Survey of published literature regarding transcription regulatory roles of NME2**

A gene expression regulatory role for NME2 (alternatively known as NM23 H2) has been questioned based on observations that it did not have a distinctly disjoint DNA binding and trans-activation domains, and it interacted with single stranded oligonucleotides rather non-specifically (1;2).

However, several lines of experiments have subsequently shown that NME2 does possess a domain specific organization relevant for transcription and shows preference for gunaine-rich sequences in target promoters suggesting a definitive transcriptional role for NME2. Some of the related observations are summarized here:

(A) Screening of cervical cancer complementary DNA (cDNA) library with a DNA fragment corresponding to a part of c-MYC promoter region identified NME2 as a factor which specifically associated with the promoter (3). Notably, the purine rich nuclease hyper sensitive segment (NHE III_1_) used in the study has been known as the major transcriptional regulatory element within c- MYC promoter (3;4) . Investigation of DNA binding properties including affinity and specificity of NME2 were probed using electrophoretic mobility shift assays (EMSAs). NME2 did not bind to unrelated DNA sequence or NHE III_1_ DNA with deletions in guanines confirming the specificity of NME2-DNA interaction. Use of RNAse protection assays showed that NME2 facilitated transcription from major promoters of c-MYC demonstrating transcriptional competence in vitro.

(B) NME2 trans-activated c-MYC promoter in chloramphenicol acetyltransferase (CAT) reporter assays (5). Taken together with previous report, this study suggested that NME2 not only associated with promoter region of c-MYC but also functionally regulated the promoter activity.

(C) Mutation of amino acid residues critical for DNA binding showed that only DNA binding was compromised without altering nucleoside diphosphate kinase activity (6). This indicated that DNA binding and enzymatic functions of NME2 reside in distinct domains indicating functional modularity.

(D) NME2 bound to translocated c-MYC promoter in Burkitt lymphoma cells (7). In vivo DMS footprinting coupled with methylation interference analysis demonstrated NME2 binding to c-MYC promoter and identified key guanine residues necessary for NME2-DNA interaction. Furthermore, EMSA, EMSA with antibody and UV cross linking coupled to Western blotting confirmed NME2 binding to c-MYC promoter.

(E) A search for guanine(G)-rich DNA sequences that might act as potential NME2 binding sites identified several candidate gene promoters such as Myeloperoxidase (MPO), cluster of differentiation molecule 11 b (cd11b), chemokine receptor 5 (CCR5), and platelet derived growth factor A (PDGF-A) (8). Luciferase reporter assays with G-rich sequences from respective promoters in presence of NME2 showed induced reporter activity. Deletion of NME2 binding site led to loss of trans-activation indicating requirement of NME2 for transcriptional induction.

(F) Interestingly, human NME2 was also shown to bind to yeast HIS4 promoter in a sequence specific manner suggesting that regulatory function may be conserved across evolutionary time scale (9).

(G) Loss of nuclear localization of NME2 associated with decreased c-MYC expression commencing the process of differentiation to specific lineages in embryonic stem cells (10).

(H) Nuclear presence for NME2 was observed in several cell types including breast cancer MDA-MB-231 cells (11).

(I) Studies in this laboratory have shown association of NME2 to c-MYC promoter in multiple cancer cell lines including lung adenocarcinoma, A549, and breast carcinoma, MDA-MB-231 (12). Using a combination of chromatin immunoprecipitation, luciferase reporter assays, qRT PCR, and RNA interference, NME2 interaction with and trans-activation of c-MYC promoter was demonstrated.

Occasionally, NME2 has also been observed as part of transcriptional co-activator complex such as OCA-S (13). Independently, yeast two hybrid screening identified NME2 interacted with ER beta and induced transcription of target genes in response to estrogen (14). Taken together, two distinct modes of gene regulatory activity of NME2 can be envisaged: one based on direct interaction with target promoters, and second as a co-factor.

A summary of the above discussion is represented as a scheme in figure below.


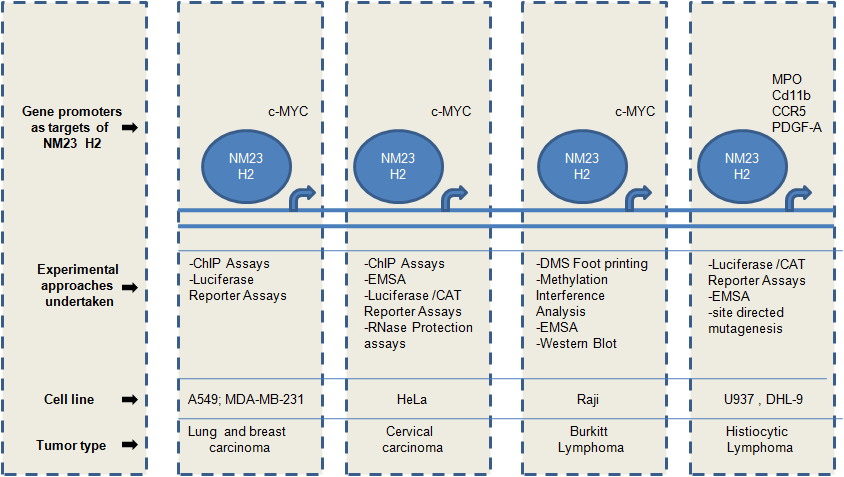


**Scheme A: The regulatory activity of NME2 (alternatively known as NM23 H2) in gene expression.** Noted across diverse cell types using multiple experimental approaches, majority of studies so far suggested a definite role for NME2 in regulation of c-MYC promoter activity (references duly provided and discussed in the text). NME2 mediated control of MPO, cd11b, CCR5, and PDGF-A promoter activity has been noted in U937 cells.


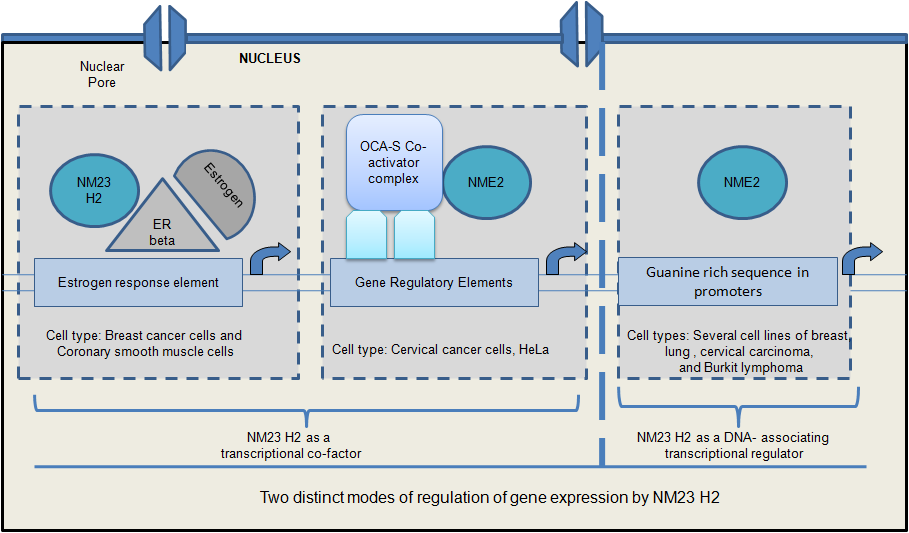


**Scheme B: NME2 (alternatively known as NM23 H2) as a regulator of gene expression.** NME2 was shown to act either as a transcriptional co-factor or directly associate with promoters to regulate outcome of gene expression. Recent reports indicated NME2 could be selectively localized to nucleus upon induction by estrogen, interact with estrogen receptor beta, and act synergistically with latter to control estrogen dependent transcription. Independently, NME2 was shown to be a part of a multi subunit co-activator complex, OCA-S on histone 2B promoter. In contrast, most studies demonstrated association of NME2 with target promoters through a guanine rich sequence.

**Supplementary Tables**

**Table S1**

**A list of metastasis suppressor genes (MSGs)**

| **S No.** | **Gene Symbol** | **Gene Name** | **Molecular Function of the protein** |
| --- | --- | --- | --- |
| 1 | BRMS1 | Breast Cancer Metastasis Suppressor 1 | A member of mSin3a family of HDAC |
| 2 | CASP8 | Caspase 8 | Cell death in response to FAS and other stimuli |
| 3 | CDH1 | E-Cadherin | Ca2^+^ dependent cell-cell adhesion |
| 4 | CDH2 | N-Cadherin | Ca2^+^ dependent cell-cell adhesion |
| 5 | CDH11 | Cadherin 11 | Ca2^+^ dependent cell-cell adhesion |
| 6 | CD44 | CD44 | Maintenance of stemness |
| 7 | CLDN1 | Claudin 1 | A component of tight junction |
| 8 | CLDN4 | Claudin 4 | A component of tight junction |
| 9 | MED23/CRSP3/DRIP130 | Vitamin D Regulatory Interacting Protein 130 | Transcriptional co-factor |
| 10 | CRMP1 | Collapsin response mediator protein 1 | Semaphoring induced signaling pathway |
| 11 | CTGF | Connective tissue growth factor | Secreted mitogen |
| 12 | DCC/Frazzled | Deleted in colon cancer | Induction of apoptosome |
| 13 | DLC1 | Deleted in liver cancer 1 | Regulation of GTPase activity |
| 14 | DRG1 | Developmentally regulated GTP binding protein 1 | P53 & PI3K/PTEN pathways |
| 15 | GAS1 | Growth Arrest Specific 1 | Blockage of entry into S-phase |
| 16 | GSN | Gelsolin | Cytoskeletal structure rearrangement |
| 17 | HUNK | Hormonally Upregulated Neu-associated Kinase | Protein kinase |
| 18 | CD82/KAI1 | CD82/Kang-Ai1 | Induction of apoptosis |
| 19 | KISS1 | KiSS-1 metastasis suppressor | G-protein coupled receptor signaling |
| 20 | KISS1R | KiSS-1 receptor | G-protein coupled receptor |
| 21 | KLF17 | Kruppel-like factor 17 | Transcriptional regulation of ID1 |
| 22 | KDM1A/LSD1 | Lysine (K)-specific demethylase 1A | Chromatin remodeling |
| 23 | MAP2K4 | Mitogen Activated Protein Kinase Kinase 4 | Protein kinase activity |
| 24 | MAP2K6 | Mitogen Activated Protein Kinase Kinase 6 | Protein kinase activity |
| 25 | MAP2K7 | Mitogen Activated Protein Kinase Kinase 7 | Protein kinase activity |
| 26 | MAPK14 | Mitogen Activated Protein Kinase 14 | Protein kinase activity |
| 27 | NME1/NM23 H1 | Non Metastatic 1 | Histidine kinase activity |
| 28 | NME2/NM23 H2 | Non Metastatic 2 | Gene regulatory activity |
| 29 | GPR68/OGR1 | G-protein coupled receptor 68 | G protein coupled receptor activity |
| 30 | RECK | Reversion-inducing-cysteine-rich protein with kazal motifs | Negative regulator of MMP-9 |
| 31 | ARHGDIB/RhoGDI2 | Rho GDP dissociation inhibitor (GDI) beta | Regulation of Rho GTPase activity |
| 32 | PEPB1/RKIP | Phosphatodylethanolamine binding protein 1 | Inhibition of RAF1 & MEK interaction |
| 33 | RRM1 | Ribonucleotide Reductase M1 | Regulation of PTEN expression & FAK phosphorylation |
| 34 | SMAD7 | SMAD family member 7 | TGF beta signaling |
| 35 | AKAP12/SSeCKS | A Kinase (PRKA) anchor protein 12 | Scaffold protein for Protein Kinase A and C |
| 36 | TXNIP | Thioredoxin Interacting Protein 1 | Transcriptional co-factor |

|  | **MICROARRAY GENES VALIDATED** | **FORWARD PRIMER SEQUENCE** | **REVERSE PRIMER SEQUENCE** | **Fold**  **Change** |
| --- | --- | --- | --- | --- |
| 1 | *TSPAN13* | ACGGCAAGTGCTCGAAATG | CTTCGGAACCCACAGCAGTT | 1.6 |
| 2 | *ANK2* | CCTTGGGCCTGTGATCGT | GCGCAGGACCACCAGTTC | 1.35 |
| 3 | *SPG21* | CAGTGCCGAGGAGCTTGAG | TACTGCTCCTCCTGGCTGATG | -1.06 |
| 4 | *HIST1H2BD* | AACGCTACGATGCCTGAACCT | CTTCTTGGAGCCCTTCTTTGG | -1.02 |
| 5 | *CD55* | CCTGGGTTCAAGCGATCCT | CACGTGTGCCCTGTAGTACCA | 1.4 |
| 6 | *FRMD6* | TCCTGCAGGGCGTGTGAT | CAGGTTCCCAGCACTCCAA | 2.26 |
| 7 | *FAS* | AACTTGGAAGGCCTGCATCA | CATCTGGAGGACAGGGCTTATC | -1.03 |
| 8 | *C20ORF177* | AGATGATGACAGCTGGGGAG | CTCGTCACGTGTCCGCT | -1.4 |
| 9 | *C3ORF38* | CTCGGTGGACTTTTCTACGC | GGTGCAGCCTCAGGACC | -1.46 |
| 10 | *SEC24A* | GCTGCCTTTAAGCTGATGTCT | GTTTGGTTCCTCTCGTGGTTT | 1.34 |
| 11 | *PPMB1* | ACGTAAACCATTCCCAGCAC | ACATCTTCTCCCTCCCTTGC | 1.07 |
| 12 | *CDS2* | TCACTGTCCGATGCAGTCTC | GGATGACAGAGCTGAGGCAG | 1.09 |
| 13 | *CHD9* | GAACCATCGCTCTTCTTGGA | CGGAGTTGGCATTCATCATT | 1.5 |
| 14 | *CYP2S1* | CTCCCCGTTGGAGAAGAAA | GGTCAGGCTGAGGAGTTCAG | -1.1 |
| 15 | *FGA* | GACTTTGCGCTTCAGGACTT | TGGAAATTTTGAGAGGCGAT | -1.2 |
| 16 | *GOLGA7* | CATTCTGCTCTTGAATGTATTTGG | TCTCGAAGGTTGTTTGGCTT | -1.2 |
| 17 | *KDELR3* | GTTGGTGAACAGGTCCAGGT | ATCTGGAGGTCCAAGTGCTG | 1.3 |
| 18 | *PLEKHB2* | TCTTCCAGCGCTTCAAAATA | GTCTTGGTGTTCTCCACGCT | -1.8 |
| 19 | *SLC4A7* | CGTGAAGAGAGATCATGGGGAA | AGATGGACGGGAATCTCCTT | 1.1 |
| 20 | *TMED4* | AGTCTGAGCAATGGACCACC | TCAGAAGGAGCAGGATTACCA | -1.1 |
| 21 | *TOM1L1* | GCATCTCCCGACCTGTTTTA | TGATGTCCGCCATATTGATG | 1.02 |
| 22 | *TPM1* | CCAACTCTTCCTCAACCAGC | CTCAAAGATGCCCAGGAGAA | 3.6 |
| 23 | *ZDHHC2* | ACACAACTTGTTCGCCAGTG | GTGTTCATCACCCTCCTGCT | 1.4 |
| 24 | *RHOB* | GGGACAGAAGTGCTTCACCT | CGACGTCATTCTCATCTGCT | -1.03 |
| 25 | *HIST1H2AC* | GATTGCAATGTCTGGACGTG | CGTAGTTGCCTTTACGGAGC | -2.08 |
| 26 | *KYNU* | ATGCGACAGGAATATGAACCAA | GATAGCCCCAAGGACAAAGAGT | -1.3 |
| 27 | *MAML1* | GAAGGGATAGCATTGGGAGATA | TGGCAGGGTGGCAGGATT | -1.14 |
| 28 | *LRRC8* | AGGCTTTGTTGTTCTGATGTT | TAGACGGTGCTTTGTATGTTTAGG | 1.3 |
| 29 | *CTGF* | TTGGCCCAGACCCAACTATG | CAGGAGGCGTTGTCATTGGT | 1.3 |
| 30 | *GTF2E2* | GAAATACCCTGTCGCTTCAGA | AGCTGTCAGTTTTCTGTGGATG | -1.6 |
| 31 | *NME2* | CTGTCTTCACCACGTTCAGC | GGCCTCTGAAGAACACCTGA | -3.0/-50.0 |
| 32 | *Beta actin* | TGCGTGACATTAAGGAGAAG | CTGCATCCTGTCGGCAATG | NA |
| 33 | *MYC* | TGAGGAGACACCGCCCAC | CAACATCGATTTCTTCCTCATCTTC | -1.9 |
| 34 | *NME1* | CTGCAGCCGGAGTTCAAC | GCAATGAAGGTACGCTCACAGT | -12 |
| 35 | *B2M* | TGCTGTCTCCATGTTTGATGTATCTCT | TCTCTGCTCCCCACCTCTAAG | NA |

**Table S2 Primer information for gene expression microarray validation**

Sequences of primers used for validating gene expression microarray following transient depletion of *NME2*. B2M, Beta actin are endogenous controls. NME2 level was decreased 3 fold in partially depleted (Pd-NME2) and 50 fold in totally depleted (Td-NME2) A549 cells.

**Table S3**

**Sequences of primers used for validating ChIP-chip peaks**

| **GENES VALIDATED**  **FROM ChIP-chip LIST** | **FORWARD PRIMER SEQUENCE** | **REVERSE PRIMER SEQUENCE** |
| --- | --- | --- |
| *ASH2L* | AGTGGGTTTTAGAAATGCTG | CAGCAGATGTTGCTACCAAG |
| *BTG1* | TTGAATAGGTAAAAGCTGCC | GCTGTGGCTTGCTTATTGTC |
| *ELK3* | AAAAAGGAAGGAAGGCCAGG | CATCTTGCTATGTTGCCCAG |
| *ERBB4* | TATGCCACTTTCCCCTGAAC | TGGCTGTGTGTTCCCAACTA |
| *FUBP1* | TAGAGTGGCTCTAAGGCTGG | GTGGCAACCATTATGCAATC |
| *HNF4G* | GATTGCTTGAGCTCAGGAGG | AAGTAGAACCTAGACCCCTG |
| *INHBA* | TAGAGCCTTGGAAAGATGGG | ACCAGATCACAGAGAGGTAG |
| *KLF10* | TTGCTTTGACCGGGAGAATG | TCTGCCTTGTGGCTAGTTTC |
| *KYNU* | ATGCGACAGGAATATGAACCAA | GATAGCCCCAAGGACAAAGAGT |
| *LRRC8* | AGGCTTTGTTGTTCTGATGTT | TAGACGGTCCTTTGTATGTTTAGG |
| *MAML* | TAGTTCCTGACCAGCCTAAC | CAACAGGGCAAGACTCCATC |
| *MSH6* | TACCCTTGTTTAAGCGACCC | CGTCATGCTTTTCTAACCAG |
| *RNF6* | TAAACCAAACGTAAAAACCTAAGT | GCTGCGCAAGTCTGATT |
| *SQLE* | GAGAGGGGGAGAACAACAAC | AGAAAAAGAAAAAGCCAGAGTAAT |
| *STK4* | AAGTCTTCTGGGAAGAGGTG | GGTGTGAGATTTCATTTTGGG |
| *TIA* | ACCCTCATGTTTGGTCAAGG | CTCACTAATACACCCCATCC |
| *TSPAN13* | ATTGGAAATTCACGGGACAA | CATGGCACATTTTCAGCATC |
| *UGT2B7* | TATATTTGGCTTGTAGACC | GAAGCATCACTCCCTGTTG |
| *RHOB* | GGGACAGAAGTGCTTCACCT | CGACGTCATTCTCATGTGCT |
| *CTGF* | TGGGGACACCCAATCAATAG | GAATGATGGGGTCTGGCTTA |
| *VCL* | CAGCCGGCTCCAGCGG | GCAGCTCAGACCTGGG |
| *PDCD6* | TGAGGCCAGGAGTTTAAGAC | GTTCAAGAGATTCTCCTGCC |
| *PDCL3* | ATGAGGAGCTCAGGAGTTCG | TCAAGTGATTCTCCTGCCTC |
| *PTPRC* | AATCAGCAAAGTGGGGACAG | TGGACTCAAGAAGTCCCACCTG |

**Supplementary materials and methods**

**Generation of stable NME2-depleted, and NME, vinculin double knockdown clones of A549 cells, NME1 silencing and viability analysis**

A549 cells were transfected using HuSH pRS (Origene Inc.) plasmid vector containing a U6 shRNA expression cassette consisting of a 29 bp NME2 specific sequence, and stable clones were puromycin selected.

We used commercially available shRNAs against human NME2 as provided by Origene Inc. USA (catalogue no. TR311160). The shRNA sequences are provided below:

-AGAACACCTGAAGCAGCACTACATTGACC (provided 50 fold decrease in NME2 mRNA level)

-CGTGGTGAAGACAGGCCGAGTGATGCTTG (provided 3 fold decrease in NME2 mRNA level)

Two additional shRNAs (from catalogue no. TR311160) were used for transient silencing of NME2 in H157 cell line (supplementary figure S8).

-GAGACCAATCCAGCAGATTCAAAGCCAGG

-AGCCTGAAGAACTGGTTGACTACAAGTCT

We generated clones of lung adenocarcinoma A549 cells with stable depletion of NME2. Two clones of A549 cells had stable depletion of NME2: first, partially depleted NME2 cells (<3-fold reduced level of NME2) and, second, almost totally depleted NME2 cells (<50-fold reduced level of NME2). A third clone had scrambled non-specific shRNA and served as control cells. These cells were used for tail-vein injection in nude mice.

Additionally, we generated several clones of A549 cells with stable double knockdown of NME2 and vinculin. We used pLKO.1 based lentiviral vector for NME2 knockdown, and pGIPZ based lentiviral vector for vinculin knockdown. The clone with best knockdown of both NME2 and vinculin was chosen for metastasis analysis in zebrafish.

NME1 was silenced using siRNA from Santa Cruz Biotechnology, CA, USA (catalogue no. sc-29414). This is a pool of 3 target specific siRNAs. The viability analysis of cells was done using luminescent CellTiter-Glo Assay from Promega, Madison, USA.

**Recombinant protein purification**

Recombinant NME1 protein was purchased from Origene Inc. USA; catalogue no. TP301731. Recombinant histidine tagged NME2 was expressed in E. coli using pRSETA-NME2 and purified using Ni-NTA chromatography to obtain His-tagged protein. Subsequently, histidine tag from recombinant protein was removed, resin bound protein was cleaved using enterokinase [(0.6 mg per25 mg of fusion protein in reaction buffer (50mM TrispH 8.0, 5mM CaCl2)]. Enterokinase was removed using enterokinase removal kit.

**Direct binding assay**

Oligonucleotide pull down assay was used to probe direct binding of NME2 and NME1 with biotinylated oligonucleotides from vinculin and c-myc promoter. For pull down assays, 1 micro gram of biotinylated wild type NME2 motif and as well as its mutant motif were incubated with 1 micro gram of recombinant/purified NME2 and NME1 for 1 hour at room temperature in binding buffer (15% Glycerol, 12mM HEPES, 4mM Tris, 150mM KCl, 1mM EDTA and 1mM DTT). The whole complex was incubated with streptavidin-agarose (Thermo Fisher catalogue no. 20349) for 4 hours at 4 degree temperature. In all experiments, the beads were washed three times with washing buffer (50 mM Tris-HCl [pH 7.5] and 150 mM NaCl) and the bound proteins were eluted by boiling in 5× sodium dodecyl sulfate (SDS) sample buffer (20 mM Tris-HCl [pH 6.8], 10% glycerol, 4% SDS, 100 mM dithiothreitol, 4 mM EDTA, and 0.025% Coomassie brilliant blue R250) and were subjected to western blot analysis using antibody from Abcam, Cambridge, UK (Abcam - ab60602) which recognizes both NME1 and 2. The wild type sequence of oligonucleotide from vinculin promoter is the following: biotin-5’-CTCCTTATAAG^*^G^*^GCATAATGGGAGCGGAGCGG; the sequence which is underlined represents 12-mer NME2 motif; the bases marked by * were mutated. Mutant oligonucleotide sequence: biotin- 5’-CTCCTTATAAAAGCATAATGGGAGCGGAGCGG; mutant/disrupted oligonucleotide served as binding control. Additional bases flanking 12-mer motif were added to allow for efficient biotinylation.

**Cell invasion, anoikis assays, VCL promoter luciferase activity, and Silencing of VCL**

Invasion potential of cells and anoikis was determined in a variation of Boyden chamber assay using Cell bio Labs CytoSelect TM invasion and Cell Biolabs CytoSelect TM anoikis assay kit respectively as per manufacturer’s instructions. Briefly, we suspended cells to a suggested density (~0.5 x 10^6^ cells/ml in serum free media) and incubated for 24 hours in a cell culture incubator for invasion assays. The upper surface of the insert membrane is coated with a uniform layer of dried basement membrane matrix solution containing extracellular matrix extracts isolated from mouse EHS sarcoma tissue, ECM gel contains laminin as a major component, collagen type IV, heparan sulfate proteoglycan, entactin and other minor components.

We used 1.1 kb minimal promoter region of VCL cloned in luciferase reporter vector (SwitchGear genomics, CA, USA) for analyzing transcriptional effect of NME2. Flag tagged NME1 was expressed in cells using vector pFLAG-CMV4 (from Addgene Inc., plasmid id: 25000).

We used a pool of 4 (four) different siRNAs targeting different regions of VCL mRNA (siRNA smart pool against VCL available from Dharmacon, USA). The collective application of 4 siRNAs ensures that effects are unlikely to be the experimental artifacts. The details of siRNA sequences and a map of VCL mRNA wherein these siRNAs bind are provided below (binding sites are underlined). Antibody against phospho-paxillin (Tyr 118) was from Cell Signaling technologies, Inc., Danvers, MA, USA.

siRNA against VCL mRNA:

1) ON-TARGETplus SMARTpool siRNA J-009288-05, VCL binds to exon 6 within VCL cDNA.

UGAGAUAAUUCGUGUGUUA

TGAGATAATTCGTGTGTTA

2) ON-TARGETplus SMARTpool siRNA J-009288-06, VCL binds to exon 21 within VCL cDNA.

GAGCGAAUCCCAACCAUAA

GAGCGAATCCCAACCATAA

3) ON-TARGETplus SMARTpool siRNA J-009288-07, VCL binds to exon 20 within VCL cDNA.

GCCAAGCAGUGCACAGAUA

GCCAAGCAGTGCACAGATA

4) ON-TARGETplus SMARTpool siRNA J-009288-08, VCL binds to boundary of exon 2-3 within VCL cDNA.

CAGCAUUUAUUAAGGUUGA

CAGCATTTATTAAGGTTGA

**VCL cDNA sequence:**

ATGCCAGTGTTTCATACGCGCACGATCGAGAGCATCCTGGAGCCGGTGGCACAGCAGATCTCCCACCTGG
TGATAATGCACGAGGAGGGCGAGGTGGACGGCAAAGCCATTCCTGACCTCACCGCGCCCGTGGCCGCCGT
GCAGGCGGCCGTCAGCAACCTCGTCCGGGTTGGAAAAGAGACTGTTCAAACCACTGAGGATCAGATTTTG
AAGAGAGATATGCCACCAGCATTTATTAAGGTTGAGAATGCTTGCACCAAGCTTGTCCAGGCAGCTCAGA
TGCTTCAGTCAGACCCTTACTCAGTGCCTGCTCGAGATTATCTAATTGATGGGTCAAGGGGCATCCTCTC
TGGAACATCAGACCTGCTCCTTACCTTCGATGAGGCTGAGGTCCGTAAAATTATTAGAGTTTGCAAAGGA
ATTTTGGAATATCTTACAGTGGCAGAGGTGGTGGAGACTATGGAAGATTTGGTCACTTACACAAAGAATC
TTGGGCCAGGAATGACTAAGATGGCCAAGATGATTGACGAGAGACAGCAGGAGCTCACTCACCAGGAGCA
CCGAGTGATGTTGGTGAACTCGATGAACACCGTGAAAGAGTTGCTGCCAGTTCTCATTTCAGCTATGAAG
ATTTTTGTAACAACTAAAAACTCAAAAAACCAAGGCATAGAGGAAGCTTTAAAAAATCGCAATTTTACTC
TAGAAAAAATGAGTGCTGAAATTAATGAGATAATTCGTGTGTTACAACTCACCTCTTGGGATGAAGATGC
CTGGGCCAGCAAGGACACTGAAGCCATGAAGAGAGCATTGGCCTCCATAGACTCCAAACTGAACCAGGCC
AAAGGTTGGCTCCGTGACCCTAGTGCCTCCCCAGGGGATGCTGGTGAGCAGGCCATCAGACAGATCTTAG
ATGAAGCTGGAAAAGTTGGTGAACTCTGTGCAGGCAAAGAACGCAGGGAGATTCTGGGAACTTGCAAAAT
GCTAGGGCAGATGACTGATCAAGTGGCTGACCTCCGTGCCAGAGGACAAGGATCCTCACCGGTGGCCATG
CAGAAAGCTCAGCAGGTATCTCAGGGTCTGGATGTGCTCACAGCAAAAGTGGAAAATGCAGCTCGCAAGC
TGGAAGCCATGACCAACTCAAAGCAGAGCATTGCAAAGAAGATCGATGCTGCTCAGAACTGGCTTGCAGA
TCCAAATGGTGGACCGGAAGGAGAAGAGCAGATTCGAGGTGCTTTGGCTGAAGCTCGGAAAATAGCAGAA
TTATGTGATGATCCTAAAGAAAGAGATGACATTCTACGTTCCCTTGGGGAAATATCTGCTCTGACTTCTA
AATTAGCAGATCTACGAAGACAGGGGAAAGGAGATTCTCCAGAGGCTCGAGCCTTGGCCAAACAGGTGGC
CACGGCCCTGCAGAACCTGCAGACCAAAACCAACCGGGCTGTGGCCAACAGCAGACCGGCCAAAGCAGCT
GTACACCTTGAGGGCAAGATTGAGCAAGCACAGCGGTGGATTGATAATCCCACAGTGGATGACCGTGGAG
TCGGTCAGGCTGCCATCCGGGGGCTTGTGGCCGAAGGGCATCGTCTGGCTAATGTTATGATGGGGCCTTA
TCGGCAAGATCTTCTCGCCAAGTGTGACCGAGTGGACCAGCTGACAGCCCAGCTGGCTGACCTGGCTGCC
AGAGGGGAAGGGGAGAGTCCTCAGGCACGAGCACTTGCATCTCAGCTCCAAGACTCCTTAAAGGATCTAA
AAGCTCGGATGCAGGAGGCCATGACTCAGGAAGTGTCAGATGTTTTCAGCGATACCACAACTCCCATCAA
GCTGTTGGCAGTGGCAGCCACGGCGCCTCCTGATGCGCCTAACAGGGAAGAGGTATTTGATGAGAGGGCA
GCTAACTTTGAAAACCATTCAGGAAAGCTTGGTGCTACGGCCGAGAAGGCGGCTGCGGTTGGTACTGCTA
ATAAATCAACAGTGGAAGGCATTCAGGCCTCAGTGAAGACGGCCCGAGAACTCACACCCCAGGTGGTCTC
GGCTGCTCGTATCTTACTTAGGAACCCTGGAAATCAAGCTGCTTATGAACATTTTGAGACCATGAAGAAC
CAGTGGATCGATAATGTTGAAAAAATGACAGGGCTGGTGGACGAAGCCATTGATACCAAATCTCTGTTGG
ATGCTTCAGAAGAAGCAATTAAAAAAGACCTGGACAAGTGCAAGGTAGCTATGGCCAACATTCAGCCTCA
GATGCTGGTTGCTGGGGCAACCAGTATTGCTCGTCGGGCCAACCGGATCCTGCTGGTGGCTAAGAGGGAG
GTGGAGAATTCCGAGGATCCCAAGTTCCGTGAGGCTGTGAAAGCTGCCTCTGATGAATTGAGCAAAACCA
TCTCCCCGATGGTGATGGATGCAAAAGCTGTGGCTGGAAACATTTCCGACCCTGGACTGCAAAAGAGCTT
CCTGGACTCAGGATATCGGATCCTGGGAGCTGTGGCCAAGGTCAGAGAAGCCTTCCAACCTCAGGAGCCT
GACTTCCCGCCGCCTCCACCAGACCTTGAACAACTCCGACTAACAGATGAGCTTGCTCCTCCCAAACCAC
CTCTGCCTGAAGGTGAGGTCCCTCCACCTAGGCCTCCACCACCAGAGGAAAAGGATGAAGAGTTCCCTGA
GCAGAAGGCCGGGGAGGTGATTAACCAGCCAATGATGATGGCTGCCAGACAGCTCCATGATGAAGCTCGC
AAATGGTCCAGCAAGGGCAATGACATCATTGCAGCAGCCAAGCGCATGGCTCTGCTGATGGCTGAGATGT
CTCGGCTGGTAAGAGGGGGCAGTGGTACCAAGCGGGCACTCATTCAGTGTGCCAAGGACATCGCCAAGGC
CTCAGATGAGGTGACTCGGTTGGCCAAGGAGGTTGCCAAGCAGTGCACAGATAAACGGATTAGAACCAAC
CTCTTACAGGTATGTGAGCGAATCCCAACCATAAGCACCCAGCTCAAAATCCTGTCCACAGTGAAGGCCA
CCATGCTGGGCCGGACCAACATCAGTGATGAGGAGTCTGAGCAGGCCACAGAGATGCTGGTTCACAATGC
CCAGAACCTCATGCAGTCTGTGAAGGAGACTGTGCGGGAAGCTGAAGCTGCTTCAATCAAAATTCGAACA
GATGCTGGATTTACACTGCGCTGGGTTAGAAAGACTCCCTGGTACCAG

**Gene expression profiling and meta-analysis**

For transcriptome analysis, total RNA was processed to hybridize to Affymetrix HG-U133 plus 2.0 GeneChip as per manufacturer’s instructions. Three biological replicates were averaged and significance analysis performed using GCOS (at least 1.2 fold, *P* <0.005 of fold change). Results were validated by quantitative real time (qRT) PCR using SYBR green chemistry on ABI 7500 Fast. GSEA was used to find similarity in gene expression pattern between lung cancer clinical sample datasets and NME2-deplted A549 cells at default settings.

**ChIP-chip assay, peak generation, motif discovery, transcription factor enrichment**

We resorted to expressing NME2 with a MYC tag and used an antibody against MYC epitope (Sigma Aldrich M4439; mouse monoclonal antibody which only recognizes c-MYC tag) to immunoprecipitate NME2 occupied DNA fragments to unambiguously (*i.e.*, exclude cross-reactivity with NME1) probe NME2 targets. Non-specific mouse IgG (catalogue no. 15381; Sigma Aldrich) was used as a mock in ChIP assays.

We have previously confirmed addition of the MYC tag does not interfere with its transcriptional activity in luciferase reporter and chromatin immunoprecipitation (ChIP) assays (12). Three independent experiments were performed, cross-linked chromatin was immunoprecipitated, sequeanase amplified, labeled, fragmented and DNA hybridized to a set of Affymetrix 1.0 R oligonucleotide microarrays wherein -7.5 to +2.5 kb of promoter regions representing 24,500 genes were tiled at 35 bp resolution (the coverage extended up to 10 kb upstream for cancer related genes) and scanned as suggested in the Affymetrix ChIP-chip protocol. Following hybridization, the signals obtained were averaged for three experiments and analyzed using ChIPOtle at its default parameters (15). The significance for a genomic region was estimated using a standard Gaussian error function (*P* < 0.001). Following peak generation by ChIPOtle, a 12 mer motif for NME2 binding was identified by Gibbs motif sampler at its default parameters. Student t-test was used for statistical analysis unless otherwise specified.

Majority of ChIP-chip peaks were mapped within - 7.5 to +2.5 kb with respect to 21,917 annotated Ensembl genes. Occasionally, as expected peaks were observed up to 10 kb upstream of TSS and are mentioned accordingly. In order to check significance of distribution of the peaks with respect to their position from TSS, 1000 promoter regions (10 kb, spanning -7.5 to +2.5 kb of TSS) were randomly selected for each observed ChIP-chip peak, from the same chromosome. Following this a sequence equal to the length of the observed peak was randomly mapped within each promoter region and position from TSS averaged across all the 1000 regions. This gave a distribution that could be observed by chance for every observed peak. Significance of actual occurrence was analyzed using two sample Kolmogorov-Smirnov test (*P* =0.006).

We considered all peaks with binding enrichment of 2 fold or more for motif discovery. The motif is represented by weblogo program (16). To probe the significance of distribution of motif within ChIP-chip identified promoters, control groups from promoter regions in the genome (excluding ChIP-chip identified promoters with peaks) were generated. The motifs actually picked up in our ChIP-chip experiment showed a difference in distribution with respect to transcription start site (two sample Kolmogorov-Smirnov test, *P*<0.01). The peak sequences were given as input into the Match tool to scan for enriched PWMs listed in TRANSFAC professional.

ChIP assays were performed as per the protocol provided by Upstate Biotechnology with modifications as suggested in Fast ChIP protocol (17). NME2 targets were validated by semi-quantitative PCR using primers flanking peak regions as obtained from ChIP-chip assay. This was done with ChIP extracts against endogenous NME2 (Santacruz biotechnology, CA, USA; catalogue no. sc-14789) in contrast to ChIP-chip experiments where NME2 was over-expressed.

**Gene expression and meta-analysis**

Expression level of each gene was quantified against a house keeping gene, B2 microglobulin (*B2M*), according to relative curve standard method (18). The expression of NME2 in 44 patient derived lung cancer samples (Origene Inc.) was also checked using qRT PCR and analyzed as mentioned above.

For meta-analysis, gene expression datasets of lung cancer patients were obtained from expO (IGC, USA, [www.intgen.org](http://www.intgen.org/)) which provides gene expression datasets of clinically annotated sets of identified tumor samples.

Here we quote from the website of expO:

“The clinically annotated exp*O* data is available to all on the Internet at the [Genomic Expression Omnibus](https://expo.intgen.org/geo/listPublicGeoTransactions.do) (GEO) and currently has 2,100 cancer associated comprehensive gene expression analyses with clinically annotated information.

The exp*O* biospecimen repository was one of the nation’s first public repositories for cancer biosamples, genomic data and clinical outcome information.  It is a national resource that is available to assist non-profit and for-profit research worldwide.  IGC performs many aspects of the project from collecting samples at the hospitals to quality control, performing DNA microarray analysis, obtaining clinical data and outcome information, and creating a public database on the [NIH GEO Website](http://expo.intgen.org/geo/listPub), to overseeing the redistribution of residual samples to allow a complete characterization of the samples to be achieved.”

Gene expressions across datasets were normalized using Z score transformation (19). GSEA was used to find similarity in gene expression pattern between lung cancer clinical sample datasets and NME2-deplted A549 cells at default settings.

The significance of changes, v (defined as relative fold change in this study) between expression pattern of clinical samples and NME2-depleted A549 cells was calculated using

v = s (1− p)

where p is a p-value calculated by performing a Student’s t-test between the advanced and early stages of cancer samples. s is the sign of the difference between the average values in these two sets considered above. v indicates the extent to which a gene is up or down-regulated in the advanced cancer stage with maximal and minimal values of 1 and -1 respectively. v for NME2-depleted A549 cells dataset was calculated similarly. Correlation coefficient was calculated between significance of changes in clinical samples and NME2-depleted A549 cell.

Additional datasets as mentioned in the main text (Fig. 1-2 and S1) were used for assessing the relationship between NME2 and survival of lung cancer patients.

**Clinical annotation of tumor specimen**

For analysis of expression of NME2, and VCL, we used commercially available lung tumor samples (cDNA qRT PCR arrays from Origene, Inc. USA). We used antibody for NME2 from Kamiya Biomedical company, USA (catalogue no. MC-412) for IHC. Antibody against vinculin was from Abcam, Cambridge, UK (catalogue no. AB-18058). All samples have been duly certified by qualified pathologists. The primary lung tumors and matched lymph node metastases for immunohistochemical analysis of NME2 and VCL were from the archives of the Anatomic Pathology of the Second University of Naples, Italy (period from 1993 to date). The details on age, sex and pathology are provided at the end of the supplementary information.

**Clinical details of 100 primary lung tumors and autologous lymph node metastases used for immunohistochemistry analyses of NME2**

**Keys:**

**Sex**

1 = Male

2 = Female

**Histology**

1 = Squamous cell carcinoma

2 = Adenocarcinoma

**Stage**

Clinical stage 1 to 4

**Immunostaining for NME2 and Vinculin**

0= No staining

1= Moderate staining

2= Intense staining


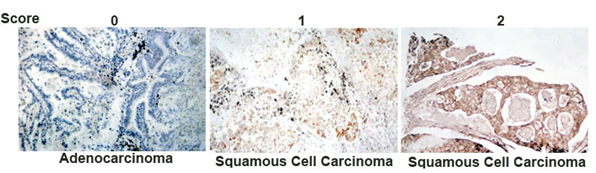


A representative example of scoring system used in immunohistochemical staining; scores 0, 1 and 2 indicate no, moderate and high staining respectively.

| Age | Sex | Histo-logy | Stage | NME2 score in primary tumor (IHC) | NME2 score in metastasis (IHC) | Vinculin score in primary tumor (IHC) | Vinculin score in metastasis (IHC) |
| --- | --- | --- | --- | --- | --- | --- | --- |
| 44 | 1 | 1 | 3 | 1 | 0 | 1 | 1 |
| 60 | 2 | 1 | 4 | 0 | 0 | 0 | 0 |
| 70 | 1 | 2 | 2 | 2 | 1 | 0 | 0 |
| 73 | 1 | 1 | 2 | 2 | 0 | 0 | 2 |
| 77 | 2 | 1 | 3 | 1 | 0 | 1 | 2 |
| 49 | 1 | 2 | 4 | 0 | 0 | 2 | 2 |
| 58 | 1 | 1 | 4 | 0 | 0 | 1 | 2 |
| 56 | 1 | 2 | 2 | 2 | 1 | 0 | 1 |
| 45 | 1 | 1 | 2 | 2 | 1 | 0 | 0 |
| 68 | 1 | 1 | 2 | 2 | 1 | 0 | 0 |
| 66 | 2 | 2 | 3 | 1 | 0 | 1 | 2 |
| 52 | 2 | 2 | 4 | 0 | 0 | 2 | 2 |
| 58 | 1 | 2 | 4 | 0 | 0 | 2 | 2 |
| 40 | 2 | 2 | 4 | 1 | 0 | 0 | 1 |
| 70 | 1 | 1 | 2 | 2 | 1 | 0 | 1 |
| 69 | 2 | 1 | 2 | 2 | 1 | 0 | 1 |
| 55 | 1 | 1 | 2 | 2 | 1 | 0 | 2 |
| 65 | 1 | 2 | 2 | 2 | 0 | 1 | 2 |
| 73 | 1 | 1 | 4 | 1 | 0 | 1 | 2 |
| 60 | 1 | 2 | 2 | 2 | 0 | 0 | 2 |
| 61 | 1 | 2 | 2 | 2 | 1 | 1 | 1 |
| 62 | 2 | 2 | 4 | 0 | 0 | 1 | 1 |
| 78 | 2 | 2 | 2 | 2 | 0 | 0 | 1 |
| 68 | 1 | 2 | 3 | 1 | 0 | 0 | 2 |
| 73 | 1 | 2 | 2 | 2 | 1 | 0 | 0 |
| 78 | 1 | 2 | 2 | 2 | 1 | 0 | 1 |
| 54 | 2 | 1 | 2 | 2 | 0 | 1 | 2 |
| 63 | 1 | 2 | 2 | 1 | 1 | 1 | 1 |
| 70 | 1 | 2 | 2 | 2 | 1 | 0 | 1 |
| 79 | 2 | 1 | 2 | 2 | 1 | 1 | 1 |
| 58 | 1 | 2 | 3 | 1 | 0 | 1 | 2 |
| 60 | 1 | 1 | 2 | 2 | 1 | 0 | 1 |
| 59 | 2 | 2 | 3 | 1 | 0 | 0 | 2 |
| 69 | 2 | 2 | 2 | 1 | 1 | 1 | 1 |
| 78 | 1 | 2 | 3 | 1 | 0 | 1 | 2 |
| 79 | 1 | 2 | 3 | 1 | 0 | 1 | 1 |
| 67 | 1 | 2 | 2 | 2 | 1 | 0 | 2 |
| 63 | 1 | 2 | 3 | 2 | 1 | 1 | 1 |
| 79 | 1 | 2 | 2 | 2 | 1 | 0 | 1 |
| 74 | 1 | 2 | 2 | 2 | 0 | 0 | 2 |
| 64 | 2 | 2 | 4 | 0 | 0 | 2 | 2 |
| 70 | 1 | 2 | 4 | 1 | 0 | 1 | 1 |
| 66 | 1 | 2 | 2 | 1 | 1 | 1 | 1 |
| 71 | 1 | 2 | 4 | 1 | 1 | 2 | 2 |
| 58 | 1 | 2 | 2 | 2 | 1 | 0 | 1 |
| 74 | 1 | 1 | 3 | 1 | 0 | 1 | 2 |
| 80 | 1 | 1 | 2 | 2 | 1 | 1 | 2 |
| 69 | 1 | 2 | 4 | 1 | 0 | 1 | 2 |
| 71 | 2 | 2 | 4 | 1 | 0 | 1 | 2 |
| 77 | 2 | 2 | 2 | 2 | 1 | 0 | 1 |
| 68 | 1 | 1 | 2 | 2 | 0 | 0 | 2 |
| 66 | 1 | 2 | 3 | 1 | 1 | 1 | 1 |
| 65 | 1 | 2 | 4 | 1 | 0 | 1 | 2 |
| 79 | 1 | 2 | 2 | 2 | 1 | 0 | 1 |
| 77 | 2 | 1 | 2 | 1 | 0 | 1 | 2 |
| 75 | 1 | 1 | 3 | 1 | 0 | 1 | 2 |
| 70 | 1 | 2 | 2 | 2 | 1 | 1 | 0 |
| 69 | 2 | 1 | 3 | 1 | 0 | 1 | 2 |
| 74 | 1 | 1 | 2 | 2 | 1 | 1 | 1 |
| 73 | 1 | 2 | 2 | 1 | 0 | 0 | 2 |
| 69 | 1 | 1 | 3 | 0 | 0 | 1 | 1 |
| 63 | 2 | 2 | 4 | 1 | 0 | 1 | 1 |
| 80 | 1 | 2 | 2 | 2 | 1 | 0 | 0 |
| 61 | 1 | 1 | 3 | 0 | 0 | 1 | 1 |
| 71 | 2 | 1 | 2 | 1 | 1 | 2 | 1 |
| 78 | 1 | 2 | 2 | 1 | 0 | 1 | 2 |
| 66 | 1 | 1 | 4 | 0 | 0 | 2 | 2 |
| 55 | 2 | 2 | 4 | 1 | 0 | 2 | 2 |
| 80 | 1 | 2 | 2 | 2 | 1 | 0 | 1 |
| 66 | 2 | 1 | 3 | 0 | 0 | 1 | 1 |
| 66 | 1 | 1 | 3 | 2 | 0 | 0 | 2 |
| 62 | 2 | 1 | 3 | 1 | 1 | 1 | 1 |
| 61 | 1 | 1 | 3 | 1 | 0 | 1 | 2 |
| 77 | 2 | 2 | 2 | 2 | 1 | 0 | 1 |
| 81 | 1 | 2 | 2 | 1 | 0 | 1 | 2 |
| 73 | 1 | 1 | 4 | 1 | 0 | 1 | 2 |
| 68 | 2 | 1 | 2 | 2 | 1 | 1 | 0 |
| 77 | 1 | 2 | 3 | 1 | 0 | 1 | 2 |
| 76 | 1 | 1 | 3 | 2 | 1 | 1 | 1 |
| 72 | 2 | 1 | 2 | 1 | 0 | 0 | 2 |
| 65 | 1 | 2 | 4 | 0 | 0 | 1 | 1 |
| 48 | 1 | 1 | 3 | 1 | 0 | 1 | 1 |
| 71 | 1 | 2 | 2 | 2 | 1 | 0 | 0 |
| 65 | 2 | 2 | 4 | 0 | 0 | 1 | 1 |
| 72 | 2 | 1 | 2 | 1 | 1 | 2 | 1 |
| 49 | 2 | 1 | 3 | 1 | 0 | 1 | 2 |
| 65 | 1 | 2 | 4 | 0 | 0 | 2 | 2 |
| 54 | 2 | 1 | 3 | 1 | 0 | 2 | 2 |
| 78 | 2 | 2 | 2 | 2 | 1 | 0 | 1 |
| 65 | 1 | 2 | 3 | 0 | 0 | 1 | 1 |
| 69 | 1 | 1 | 4 | 1 | 0 | 1 | 1 |
| 81 | 1 | 1 | 4 | 0 | 0 | 0 | 0 |
| 45 | 2 | 1 | 3 | 2 | 1 | 0 | 0 |
| 75 | 1 | 1 | 2 | 2 | 0 | 0 | 2 |
| 70 | 1 | 1 | 4 | 1 | 0 | 1 | 2 |
| 48 | 1 | 1 | 4 | 0 | 0 | 2 | 2 |
| 67 | 1 | 2 | 4 | 0 | 0 | 1 | 2 |
| 55 | 2 | 2 | 3 | 2 | 1 | 0 | 1 |
| 43 | 1 | 2 | 2 | 2 | 1 | 0 | 0 |
| 66 | 2 | 2 | 3 | 2 | 1 | 0 | 0 |

**Clinical annotations of lung tumors used for qRT PCR analysis of NME2**

| | **Sex** | **Diagnosis** | **Clinical  Stage** | **Sex** | **Diagnosis** | **Clinical  Stage** | | --- | --- | --- | --- | --- | --- | | Female | Carcinoma of lung, squamous cell | 0 | Male | Carcinoma of lung, squamous cell | IIB | | Male | Carcinoma of lung, non-small cell | 0 | Male | Adenocarcinoma of lung | IIB | | Male | Carcinoma of lung, squamous cell | 0 | Male | Carcinoma of lung, squamous cell | IIB | | Female | Adenocarcinoma of lung, bronchioloalveolar | 0 | Female | Adenocarcinoma of lung | IIB | | Female | Adenocarcinoma of lung | 0 | Female | Adenocarcinoma of lung | IIB | | Male | Malignant melanoma, metastatic | 0 | Male | Carcinoma of lung, squamous cell | IIB | | Male | Carcinoma of lung, squamous cell | 0 | Male | Carcinoma of lung, squamous cell | IIB | | Female | Adenocarcinoma of lung | 0 | Male | Carcinoma of lung, squamous cell | IIIA | | Male | Carcinoma of lung, squamous cell | IA | Female | Carcinoma of lung, adenosquamous | IIIA | | Female | Carcinoma of lung, large cell | IA | Male | Carcinoma of lung, large cell, neuroendocrine | IIIA | | Female | Adenocarcinoma of lung | IA | Male | Carcinoma of lung, small cell | IIIA | | Female | Carcinoma of lung, bronchioloalveolar, non-mucinous | IA | Male | Adenocarcinoma of lung | IIIA | | Female | Adenocarcinoma of lung | IA | Male | Carcinoma of lung, sarcomatoid | IIIB | | Male | Adenocarcinoma of lung | IA | Female | Adenocarcinoma of lung, bronchioloalveolar | IIIB | | Female | Carcinoma of lung, squamous cell | IB | Female | Carcinoma of lung, adenosquamous | IIIB | | Male | Carcinoma of lung, squamous cell | IB | Male | Adenocarcinoma of lung, bronchioloalveolar | IIIB | | Male | Carcinoma of lung, squamous cell | IB | Male | Carcinoma of lung, squamous cell | IIIB | | Female | Adenocarcinoma of lung, bronchioloalveolar | IB | Female | Adenocarcinoma of lung | IV | | Female | Carcinoma of lung, large cell, neuroendocrine | IB | Male | Carcinoma of lung, non-small cell, metastatic | IV | | Male | Carcinoma of lung, non-small cell | IB | Female | Carcinoma of lung, non-small cell, metastatic | IV | | Not Sp | Adenocarcinoma of lung | IIA | Female | Carcinoma of lung, squamous cell, metastatic | IV | | Male | Carcinoma of lung, small cell | IIA | Male | Adenocarcinoma of lung | IV | | Male | Carcinoma of lung, squamous cell | IIA | Male | Adenocarcinoma of lung, metastatic | IV | | Male | Adenocarcinoma of lung | IIB | Male | Adenocarcinoma of lung, metastatic | IV | |  |
| --- | --- | --- | --- | --- | --- | --- | --- | --- | --- | --- | --- | --- | --- | --- | --- | --- | --- | --- | --- | --- | --- | --- | --- | --- | --- | --- | --- | --- | --- | --- | --- | --- | --- | --- | --- | --- | --- | --- | --- | --- | --- | --- | --- | --- | --- | --- | --- | --- | --- | --- | --- | --- | --- | --- | --- | --- | --- | --- | --- | --- | --- | --- | --- | --- | --- | --- | --- | --- | --- | --- | --- | --- | --- | --- | --- | --- | --- | --- | --- | --- | --- | --- | --- | --- | --- | --- | --- | --- | --- | --- | --- | --- | --- | --- | --- | --- | --- | --- | --- | --- | --- | --- | --- | --- | --- | --- | --- | --- | --- | --- | --- | --- | --- | --- | --- | --- | --- | --- | --- | --- | --- | --- | --- | --- | --- | --- | --- | --- | --- | --- | --- | --- | --- | --- | --- | --- | --- | --- | --- | --- | --- | --- | --- | --- | --- | --- | --- | --- | --- | --- | --- |

Reference List

1. Hildebrandt,M., Lacombe,M.L., Mesnildrey,S. and Veron,M. (1995) A human NDP-kinase B specifically binds single-stranded poly-pyrimidine sequences. *Nucleic Acids Res.*, **23**, 3858-3864.

2. Michelotti,E.F., Sanford,S., Freije,J.M., MacDonald,N.J., Steeg,P.S. and Levens,D. (1997) Nm23/PuF does not directly stimulate transcription through the CT element in vivo. *J. Biol. Chem.*, **272**, 22526-22530.

3. Postel,E.H., Berberich,S.J., Flint,S.J. and Ferrone,C.A. (1993) Human c-myc transcription factor PuF identified as nm23-H2 nucleoside diphosphate kinase, a candidate suppressor of tumor metastasis. *Science*, **261**, 478-480.

4. Gonzalez,V. and Hurley,L.H. (2010) The c-MYC NHE III(1): function and regulation. *Annu. Rev. Pharmacol. Toxicol.*, **50**, 111-129.

5. Berberich,S.J. and Postel,E.H. (1995) PuF/NM23-H2/NDPK-B transactivates a human c-myc promoter-CAT gene via a functional nuclease hypersensitive element. *Oncogene*, **10**, 2343-2347.

6. Postel,E.H., Weiss,V.H., Beneken,J. and Kirtane,A. (1996) Mutational analysis of NM23-H2/NDP kinase identifies the structural domains critical to recognition of a c-myc regulatory element. *Proc. Natl. Acad. Sci. U. S. A*, **93**, 6892-6897.

7. Ji,L., Arcinas,M. and Boxer,L.M. (1995) The transcription factor, Nm23H2, binds to and activates the translocated c-myc allele in Burkitt's lymphoma. *J. Biol. Chem.*, **270**, 13392-13398.

8. Postel,E.H., Berberich,S.J., Rooney,J.W. and Kaetzel,D.M. (2000) Human NM23/nucleoside diphosphate kinase regulates gene expression through DNA binding to nuclease-hypersensitive transcriptional elements. *J. Bioenerg. Biomembr.*, **32**, 277-284.

9. Zimmermann,S., Baumann,A., Jaekel,K., Marbach,I., Engelberg,D. and Frohnmeyer,H. (1999) UV-responsive genes of arabidopsis revealed by similarity to the Gcn4-mediated UV response in yeast. *J. Biol. Chem.*, **274**, 17017-17024.

10. Zhu,S., Wurdak,H., Wang,J., Lyssiotis,C.A., Peters,E.C., Cho,C.Y., Wu,X. and Schultz,P.G. (2009) A small molecule primes embryonic stem cells for differentiation. *Cell Stem Cell*, **4**, 416-426.

11. Pinon,V.P., Millot,G., Munier,A., Vassy,J., Linares-Cruz,G., Capeau,J., Calvo,F. and Lacombe,M.L. (1999) Cytoskeletal association of the A and B nucleoside diphosphate kinases of interphasic but not mitotic human carcinoma cell lines: specific nuclear localization of the B subunit. *Exp. Cell Res.*, **246**, 355-367.

12. Thakur,R.K., Kumar,P., Halder,K., Verma,A., Kar,A., Parent,J.L., Basundra,R., Kumar,A. and Chowdhury,S. (2009) Metastases suppressor NM23-H2 interaction with G-quadruplex DNA within c-MYC promoter nuclease hypersensitive element induces c-MYC expression. *Nucleic Acids Res.*, **37**, 172-183.

13. Zheng,L., Roeder,R.G. and Luo,Y. (2003) S phase activation of the histone H2B promoter by OCA-S, a coactivator complex that contains GAPDH as a key component. *Cell*, **114**, 255-266.

14. Rayner,K., Chen,Y.X., Hibbert,B., White,D., Miller,H., Postel,E.H. and O'Brien,E.R. (2008) Discovery of NM23-H2 as an estrogen receptor beta-associated protein: role in estrogen-induced gene transcription and cell migration. *J. Steroid Biochem. Mol. Biol.*, **108**, 72-81.

15. Buck,M.J., Nobel,A.B. and Lieb,J.D. (2005) ChIPOTle: a user-friendly tool for the analysis of ChIP-chip data. *Genome Biol.*, **6**, R97.

16. Crooks,G.E., Hon,G., Chandonia,J.M. and Brenner,S.E. (2004) WebLogo: a sequence logo generator. *Genome Res.*, **14**, 1188-1190.

17. Nelson,J.D., Denisenko,O. and Bomsztyk,K. (2006) Protocol for the fast chromatin immunoprecipitation (ChIP) method. *Nat. Protoc.*, **1**, 179-185.

18. Larionov,A., Krause,A. and Miller,W. (2005) A standard curve based method for relative real time PCR data processing. *BMC. Bioinformatics.*, **6**, 62.

19. Cheadle,C., Vawter,M.P., Freed,W.J. and Becker,K.G. (2003) Analysis of microarray data using Z score transformation. *J. Mol. Diagn.*, **5**, 73-81.
